# The ESX-3 Secretion System Is Necessary for Iron and Zinc Homeostasis in *Mycobacterium tuberculosis*

**DOI:** 10.1371/journal.pone.0078351

**Published:** 2013-10-14

**Authors:** Agnese Serafini, Davide Pisu, Giorgio Palù, G. Marcela Rodriguez, Riccardo Manganelli

**Affiliations:** 1 Department of Molecular Medicine, University of Padova, Padova, Italy; 2 Public Health Research Institute - Rutgers, the State University of New Jersey, Newark, New Jersey, United States of America; The Catholic University of the Sacred Heart, Rome, Italy

## Abstract

ESX-3 is one of the five type VII secretion systems encoded by the *Mycobacterium tuberculosis* genome. We recently showed the essentiality of ESX-3 for *M. tuberculosis* viability and proposed its involvement in iron and zinc metabolism. In this study we confirmed the role of ESX-3 in iron uptake and its involvement in the adaptation to low zinc environment in *M. tuberculosis*. Moreover, we unveiled functional differences between the ESX-3 roles in *M. tuberculosis* and *M. smegmatis* showing that in the latter ESX-3 is only involved in the adaptation to iron and not to zinc restriction. Finally, we also showed that in *M. tuberculosis* this secretion system is essential for iron and zinc homeostasis not only in conditions in which the concentrations of these metals are limiting but also in metal sufficient conditions.

## Introduction

*Mycobacterium tuberculosis* is one of the most successful obligate human pathogens. Despite the fact that tuberculosis is a treatable disease, the length of treatment and the selection and diffusion of strains resistant to a wide set of antibiotics makes this disease still a severe problem for human health, causing more than one million deaths every year (http://www.who.int/tb/publications/global_report/en/). To improve tuberculosis control, the characterization of new potential drug targets is a critical goal. Secretion systems represent one of the emerging targets for antibacterial therapy given their surface localization [1,2] and the essentiality of several of them for viability or virulence.

The *M. tuberculosis* genome encodes four types of secretion systems [3]: the conserved essential Sec system, the Twin-arginine translocase (Tat) export system, and two specialized secretion systems: the accessory Sec A2 pathway and the recently discovered ESX pathway (also called Type VII Secretion System, T7SS), which is only found in mycobacteria and some Gram-positive bacteria [2,4]. 

Five ESX secretion systems are present in *M. tuberculosis*, ESX-1 to ESX-5 [5,6]. They are responsible for the secretion of Esx (small proteins belonging to the WXG100 family), Esp (ESX-1 secretion-associated proteins), PE (Pro-Glu motif) and PPE (Pro-Pro-Glu motif) proteins [7]. The best characterized T7SS is ESX-1 which is essential for virulence [8,9] due to its involvement in the arrest of phagosome maturation [10] and in the escape of the bacteria into the cytosol [11,12]. Another well-characterized ESX secretion system is ESX-5, which was extensively characterized in *M. tuberculosis*, where it was shown to be essential for growth [13], and in *Mycobacterium marinum* [14]. ESX-5 is involved in virulence, in the maintenance of cell wall integrity [15] and in secretion of PE and PPE proteins [15,16], two large families of secreted or cell wall-associated proteins characteristic of mycobacteria involved in virulence [17,18,19] and in modulation of the immune response [20,21,22]. Not much is known regarding the function of ESX-2 and ESX-4. These two secretion systems are known to be transcriptional regulated by the alternative sigma factor σ^M^ [23] and by WhiB5 [24]. This is interesting since WhiB5 was shown to be involved in down-modulation of the immune response, virulence and in reactivation in a mouse model, leading to the hypothesis that some antigens exported by ESX-2 or ESX-4 could have an immunosuppressing function or be involved in the mechanism necessary for reactivation [24]. 

The object of this study is ESX-3. The knowledge about the role of this T7SS in mycobacterial physiology is limited. Using a repressible promoter-system [25] we recently constructed a conditional *esx-3* mutant in *M. tuberculosis* and showed that ESX-3 is essential for growth and viability in Middlebrook 7H9 [26]. In *M. tuberculosis* the *esx-3* gene cluster is negatively regulated by high concentrations of iron and/or zinc, through the transcriptional repressors IdeR and Zur, respectively suggesting its involvement in adaptation to low zinc and/or iron conditions, while in *M. smegmatis* it is regulated only by iron through IdeR [27,28], suggesting that in this species it is only involved in adaptation to low iron conditions. 

To acquire iron mycobacteria produces siderophores (high affinity iron chelators). *M. tuberculosis* and *M. smegmatis* produce salicylate containing siderophores known as mycobactins [29,30]. Two forms of mycobactins are produced: carboxymycobactin a water soluble secreted molecule and the cell associated mycobactin which is a hydropobic molecule retained on the cell surface. In addition to mycobactins, *M. smegmatis* also produces a peptidic siderophore known as exochelin [31], which is the predominant siderophore secreted by this mycobacterium under iron limitation [32]. Siderophores avidly bind ferric iron and can effectively compete with host iron binding proteins for this metal. Fe^+3^-carboxymycobactin can transfer Fe^+3^ to mycobactin or bring it into the cell via the iron regulated transporter IrtAB [33,34]. The putative transporter encoded by *fxuABC* may transport Fe^+3^-exochelin complexes [35]. Previous work has linked the ESX-3 system with the ability of mycobacteria to adapt to iron limitation [26,36]. Morover, studies that examined a *M. smegmatis* exochelin synthesis mutant indicated an ESX-3 requirement for Fe^+3^-mycobactin utilization [36]. However, the precise role of ESX-3 in iron acquisition in *M. tuberculosis* is unknown. 

Zinc uptake systems have not been identified yet in mycobacteria. The genes encoding a putative ABC-zinc transporter system (Rv2059-Rv2060) and a zinc low-affinity transporter (Rv0106) are up regulated in an *M. tuberculosis* zur null mutant [27] suggesting their role in the uptake of this metal, however their functionality and effective role in zinc uptake have not been demonstrated yet.

The work presented here enhances the understanding of ESX-3’s role in mycobacteria. We showed that when *esx-3* expression is repressed, bacteria display severe growth defects in both high and low concentrations of iron and zinc with a more severe phenotype when the concentration of these metals is low, while the growth of a *M. smegmatis esx-3* null mutant is only affected in low iron conditions. Next, we observed that *M. tuberculosis* responds to *esx-3* down-regulation by up regulating genes normally induced during iron and zinc starvation, suggesting that in the absence of ESX-3 *M. tuberculosis* experiences iron and zinc deficiency. Finally, we confirmed the ESX-3 role in iron acquisition showing reduced Fe^3+^-carboxymycobactin (CM) uptake and reduced intracellular iron following *esx-3* repression.

## Materials and Methods

### Bacteria strains, media and growth conditions

The mycobacterial strains used in this study are listed in Table S1. The strains were routinely cultured in standard Middlebrook 7H9 and 7H10 supplemented with 0.05% Tween 80 and 0.5% and 0.2% of glycerol respectively. The *M. tuberculosis* cultures were further supplemented with 0.5% bovine serum albumin, 0.085% sodium chloride and 0.2% dextrose (ADN). 

The growth in defined iron and zinc conditions was performed in Minimal Medium (MM): 0.5% w/v KH_2_PO_4_; 0.5 % w/v asparagine; 0.2% glycerol; 0.05% tween 80; ADN 10%, pH 6.8 treated with Chelex100 (Sigma) at 4°C for 36 h, replacing Chelex 100 every 8-12 h. Chelex 100 was removed by filtration and the medium was supplemented with 40mg/L MgSO_4_; 0.1mg/L MnSO_4_ and the desired amounts of iron and zinc in the form of FeCl_3_ and ZnSO_4_, respectively: 50 µM iron and 3,67 µM zinc for complete MM (CMM); 2 µM iron and 3,67 µM zinc for low-iron MM (LIMM) or 0 µM for no iron supplemented MM (NIMM); 50 µM iron and no zinc for low-zinc MM (LZMM). When required, antibiotics were added at the following concentrations: 20 µg/ml kanamycin (Km); 20 µg/ml streptomycin (Sm); 50 µg/ml hygromycin (Hyg). 

*Escherichia coli* DH5α was routinely used in the DNA-cloning procedures. The cells were grown in Luria Bertani (LB) broth and LB-agar. When indicated, antibiotics were added at the following concentration: 50 µg/ml Km; 20 µg/ml (Sm); 200 µg/ml Hyg. 

For growth analyses, *M. tuberculosis* strains were pre-grown in rolling bottles in 20 ml of MM or Middlebrook 7H9 with or without 200 ng/ml of anhydrotetracycline (ATc) for 48 h. This concentration has been previously determined to stop the growth of the *esx-3* conditional mutant [26]. Subsequently, the cultures were diluted 1:10 in fresh medium plus or minus 200 ng/ml ATc and the growth was monitored measuring the increase in optical density (OD) at 540 nM. *M. smegmatis* strains were pre-grown overnight in 5 ml of MM or Middlebrook 7H9 in shaking cultures until late exponential phase, diluted to an OD_540_ of 0.025 and the growth was monitored for three days.

### Construction of *M. smegmatis* mutants

To create the unmarked *esx-3* deletion in *M. smegmatis*, we used the two-step cross-over strategy based on pNIL-pGOAL [37]. Plasmids and primers used for mutants construction are shown in Tables S1 and S2. DNA regions of about 1 kb flanking *msmeg_0615* and 3’ to *msmeg_0624* were amplified from *M. smegmatis* mc^2^ 155 genomic DNA using the RP1215/RP1216 and RP1217/RP1218 primer pairs. The two PCR fragments were cloned in pCRBluntII-TOPO (Invitrogen) and then subcloned together in p2NIL obtaining the plasmid pAGN25 (Figure S1). The *sacB/lacZ/hyg* cassette from pGOAL19 [37] was then cloned in the unique PacI restriction site of pAGN25 to obtain the final suicide plasmid pAGN27 (Figure S1). This plasmid was introduced by electroporation in *M. smegmatis* mc^2^155 and the transformants were selected on medium containing Hyg. Blue colonies expressing β-galactosidase were selected. The first cross over event was verified by PCR using the primer pair RP1277/1278 and RP1258/1279 flanking the homologous regions used for recombination (Figure S2A-B). Transformants were grown in medium without antibiotic to allow the second cross over event, and then selected on Middlebrook 7H10 supplemented with 8% sucrose and X-gal (40 µg/ml). Sucrose resistant and white colonies were screened by PCR using the primer pair RP1277/RP1279 flanking the deleted region to confirm *esx-3* deletion (Figure S2C). The *esx-3* null mutant was named MS190.

To generate the unmarked deletion of the *fxbA* gene *in M. smegmatis* we used the strategy showed in Figure S3. Briefly, 0.5-kb regions flanking the *fxbA* gene were amplified from *M. smegmatis* genomic DNA using the primers couples RP1261/RP1262 and RP1263/RP1264. The PCR products were then subcloned together in pCRBluntII-TOPO (Invitrogen) obtaining the plasmid pAGN29. The Hyg cassette flanked by *dif* sites from pAL70 [38] was then inserted in the unique BglII site between the two *fxbA-*flanking fragments in pAGN29 obtaining pAGN30. The cassette including the *fxbA* flanking regions and the Hyg-resistance determinant flanked by *dif* sites was extracted from pAGN30 and integrated using the recombineering technology [39] in the genomes of MS192 and MS81.1 (MS190 and mc^2^155 derivatives harboring the pJV53 vector carrying the genes encoding the two recombinases that mediate homologous recombination). Transformants were selected on Hyg and the integration was verified using the primer pair RP472/RP1308 and RP747/RP1309. Then, the transformants were grown in medium not supplemented with hygromycin to allow the excision of the Hyg cassette due to the recombination between the *dif* sequences. The excision was verified by the inability of the clones to grow in presence of Hyg and by PCR using the primer pair RP1308/RP1309. The deletion was further confirmed by sequencing. The *esx-3/fxbA* double mutant and the *fxbA* single mutant were named MS195 and MS197, respectively.

### RNA extraction

Total RNA was extracted from bacteria in early exponential phase grown in rolling bottles. Cells were harvested at 1000 g for 5 min. The total RNA was extracted as previously described [27]. Briefly, the cells were resuspended in 1 ml Trizol (Invitrogen) and added with 0.5 ml of 0.1-mm-diameter zirconia/silica beads (BioSpec Products) and disrupted with two 1-min pulses in a Mini-Beat Beater (BioSpec Products). The nucleic acids were extracted with a solution of chloroform: isoamyl alchol (24:1) and precipitated overnight at 4°C with isopropanol. After centrifugation, the pellet was washed with 75% ethanol and resuspended in water. DNA was removed treating samples with 4 units DNase (Ambion) for 1 h and the RNA was purified using the RNeasy kit (Quiagen). The treatment with DNase and the following clean up were performed twice to completely remove DNA. The concentration and purity of RNA were determined spectrophotometrically, integrity was verified on 1.3% agarose gel and by Agilent 2100 Bioanalyzer.

### cDNA labeling and microarray analysis

DNA array analysis was performed on RNA samples extracted from the *esx-3* conditional mutant TB79 and its parental strain TB38 grown in 35 ml of Middlebrook 7H9 added with 100 ng/ml of ATc for 48 h. The time and ATc concentration were chosen to ensure that TB79 cells were still actively dividing even if *esx-3* mRNA was at least 80% lower than in TB38 (data not shown). 

Fluorescently-labeled cDNA was produced as previously described [40] by reverse transcription (RT) of total RNA with Superscript II (Invitrogen LifeTechnologies) in the presence of Cy3-dUTP or Cy5-dUTP (Amersham Pharmacia) by using random hexamers to prime cDNA synthesis (Invitrogen Life Technologies). 

*M. tuberculosis* oligoarrays from the Center for Applied Genomics, International Center for Public Health (Newark, NJ, USA), consisting of 4295 70-mer oligonucleotides representing 3924 open reading frames (ORFs) from *M. tuberculosis* strain H37Rv and 371 unique ORFs from strain CDC 1551 that are not present in H37Rv were used for the hybridization.

Hybridization on DNA microarrays was performed as previously described [41]. Briefly, the microarrays were pre-hybridized in 0.1% SDS and 3% BSA, washed in water and in isopropanol and finally air-dried. Competitive hybridizations with equal amounts of purified Cy3/Cy5-labelled cDNA (approx. 60 pmol) were performed in duplicate with both dye arrangements.

Microarrays were scanned using an Agilent G2565CA microarray scanner system [with Scan Control 8.1 software (Agilent Technologies)], and fluorescence intensities of the two channels at each spot were quantified using Agilent Feature Extraction 10.1 software (Agilent Technologies).

Data were normalized with the Web-based tool DNMAD [42], using the print-tip lowess method after background subtraction. Genes significantly differentially expressed were identified using the significance analysis of microarrays (SAM) tool [43], part of the Tiger MultiExperiment Viewer package, version 4.6 (TMeV), available at http://www.tm4.org/mev/ [40].

Differently expressed genes were defined by a *q-*value equal to 0 and a fold difference ≥1.8 (induced) or ≤0.6 (repressed). Differentially regulated genes with q-value until 5.58 were included if part of an operon or regulon differentially regulated or if their up regulation was observed by quantitative real-time RT-PCR (i.e. *irtA* and *irtB*)*.*

DNA array data have been deposited in NCBI's Gene Expression Omnibus [44] and are accessible through GEO Series accession number GSE47834 (http://www.ncbi.nlm.nih.gov/geo/query/acc.cgi?acc=GSE47834).

### Quantitative real-time RT-PCR

Quantitative real-time RT-PCR to confirm alterations in the expression of genes identified by microarray was performed on RNA samples collected in the following ways: i) TB38 and TB79 grown in 35 ml of LIMM and Middlebrook 7H9 media with 100 ng/ml ATc for 48h; ii) TB38 and TB79 were pre-grown in CMM with or without 200 ng/ml of ATc, after 48h the cultures were diluted 1:10 with fresh medium containing 100 ng/ml of ATc and the RNA samples were collected after 48 h. In CMM two passes were performed since after a single pass, even if repression of *esx-3* was clear, up-regulation of the iron and zinc repressed genes was very weak.

500 ng of the total RNA were retro-transcribed using random primers and murine leucoblastoma virus retro-transcriptase (MULV) (Applied Biosystem) as previously described [27]. The RNA was denatured 2 min at 98°C and added to a solution containing 5.5 mM MgCl_2_, 1X MULV Buffer (Applied Biosystem), 250µM dNTPs, 2.5µM random hexamers, 10 units RNAse inhibitor, 31 units MULV enzyme (Applied Biosystem) in a final volume of 25 µl; the retro-transcription was carried out with the following cycle: 10 min at 25°C for random hexamers annealing, 50 min at 45°C for retro-transcription, 5 min at 95°C to inactive the retro-transcriptase.

Fifty ng of retro-transcribed RNA were used in each PCR reaction in a final volume of 25 µl. The quantitative real-time RT-PCR was performed with SYBR Green master mix 2X (Applied Biosystem) in Applied Biosystems 7700 Prism spectrofluorometric thermal cycler (Perkin-Elmer): a first cycle of denaturation at 95°C for 10 min to activate the enzyme; 40 cycles of 1 min denaturation, 30 sec annealing at 60°C, 30 sec elongation at 72°C. Fluorescence was measured during the annealing step and plotted automatically for each sample. The results were normalized to the amount of *sigA* mRNA [45], whose expression levels are not affected by the concentrations of intracellular iron or zinc (data not shown). RNA samples that had not been reverse transcribed were included in all experiments to exclude significant DNA contamination. For each sample, melting curves were performed to confirm the purity of the PCR products.

The fold induction was showed as ratio between *sigA* cDNA-normalized cDNA levels of: i) conditional mutant/parental strain both grown with ATc; ii) conditional mutant grown with ATc/conditional mutant grown without ATc. The primers used in these experiments are listed in Table S3.

### Streptonigrin (STN) sensitivity assay

*M. tuberculosis* was grown in Middlebrook 7H9 in rolling bottles with or without 100 ng/ml ATc for 72 h. After the first 24 h the cultures were divided in two tubes and 5 µg/ml of STN was added to one tube. After 72 h of incubation the viability of both cultures was determined by plating onto Middlebrook 7H10 plates. 

*M. smegmatis* was grown in LIMM overnight, the cultures were diluted to OD_600_ of 0.1 in 20 ml of same type of medium and incubated with STN 5 µg/ml for 30 min. Viability, determined by plating serial dilution of the cultures on Middlebrook 7H10 plates, was evaluated at time 0 and 30 minutes.

### Preparation of ^55^Fe- carboxymycobactin and iron uptake assays

 Ferri-carboxymycobactin (Fe^+3^-CM) labeling and iron uptake was performed as previously described [34]. Briefly, purified Fe-CM was deferrated by incubation with one volume of 50mM EDTA, pH 4.0 for 18 h or until the absorbance at 450 nm decreased to less than 10% of its initial value. The solution was then extracted into chloroform, the chloroform evaporated, and the residue resuspended in 50% ethanol. Desferri-CM was then mixed with ^55^FeCl_3_ (PerkinElmer) in a ratio of 2:1 CM to iron, and the mixture was incubated 10 min at room temperature. ^55^Fe^3+^-CM was then extracted into chloroform and washed twice with water to remove free unbound ^55^Fe^3+^. The chloroform was evaporated, and ^55^Fe^3+^-CM was suspended in 50% ethanol and kept in small aliquots at -20°C.

For iron uptake experiments mycobacterial strains were iron depleted by pre-growing them in NIMM for two generations and then in the presence or absence of 200 ng/ml ATc for two more generations before assessing iron uptake. At this time the cells with and without ATc were replicating at the same rate. Cultures were collected by centrifugation, diluted to 2X10^8^ bacilli per ml in NIMM and 1 ml of cells the cell suspension was incubated with ^55^Fe^+3^-CM (10^6^ cpm) at 0 or 37°C. Samples of 0.5 ml were removed at indicated time points, immediately placed in ice and uptake stopped with cold 0.1 M LiCl. Bacteria were collected by centrifugation and washed twice with cold NIMM. The bacterial pellet was resuspended in scintillation fluid and radioactivity determined using a Beckman LS6500 scintillation counter set at the wide-open window setting. The data are expressed as cpm incorporated by 1X10^8^ cells at each time point after subtracting the radioactivity incorporated in cells incubated on ice, which was less than 10% of total incorporation.

### Growth in minimal medium with hemin as iron source

Stock solutions 10 mM hemin (Sigma) and 50 mM of the iron chelator deferoxamine mesylate salt (DFO) (Sigma) were prepared fresh before each experiment dissolving them in 10mM NaOH and water respectively. *M. tuberculosis* strains were grown in NIMM, in NIMM supplemented with 50 µM DFO, in NIMM supplemented with 5 or 10 µM of hemin and 50 µM DFO. In these conditions the strains were pre-grown for 48 h with or without 200 ng/ml of ATc and subsequently the cultures were diluted 1:10 in fresh media with the same composition and the optical density was monitored for a week. *M. smegmatis* strains were pre-grown overnight in 5 ml of LIMM and next diluted to OD_540_ of 0.025 in NIMM supplemented as in *M. tuberculosis*. The growth was monitored for about 72 h.

### MIC determination by microplate Alamar Blue assay

*M. smegmatis* cultures were grown overnight in 5 ml of 7H9, diluted until final concentration of 10^4^ cells/ml. 5µl of antibiotic stocks were added to the first well of black 96-micro well plate (NUNC) containing 200 µl of diluted culture and 1:2 serial dilutions were performed in the next wells containing 100 µl of the same diluted culture. The microplate was incubated at 37°C for 48 h and then 10 µl of Alamar Blue dye (Invitrogen) was added to each well and analyzed by fluorescent spectrophotometer (Infinite F200-PRO Filter/Tecan) after 2 h. As a control Alamar Blue was also added to a well containing cells not treated with drugs and a well containing only medium (background value). After background subtraction the fluorescent value obtained from each well was normalized to the fluorescence value obtained from the well containing cells without antibiotic. The concentration able to reduce the fluorescence signal of 90% was considered the minimal inhibiting concentration (MIC). Each experiment was performed in duplicated.

### SDS sensitivity

*M. smegmatis* cultures were grown overnight in 5 ml of Middlebrook 7H9, diluted to an OD_600_ of 0.1 in 20 ml of Middlebrook 7H9 and incubated with 0.1% SDS for 120 min at 37°C in shaking flasks. Culture samples were taken at 0, 30, 60 and 120 min and serial dilutions plated on Middlebrook 7H10 plates for enumerating CFUs.

### THP1-derived macrophages infection

THP-1 monocytes (American Type Culture Collection) were grown in suspension at 37°C in 5% CO2 in RPMI (Gibco) supplemented with 10% (vol/vol) fetal bovine serum (FBS) (Gibco) and 50 μM β-mercaptoethanol. The infection was performed as previously described [46]. Briefly, the cells were plated in 96-well plates at a density of 7.5 × 10^4^ cells/well and incubated with 50 ng/ml of phorbol 12-myristate 13-acetate (PMA) (Sigma) for 24 h to induce the differentiation in macrophages. THP-1-derived macrophages were infected with *M. tuberculosis* at a multiplicity of infection of 1:20 (CFU:macrophages) for 90 min at 37°C with mycobacteria previously grown in Middlebrook 7H9. Cells were washed with PBS to remove extracellular bacteria, and fresh medium with or without 200 ng/ml ATc was added. The medium was replaced every 48 h. At the time 0 and every 24 h for 6 days the medium was removed and then intracellular bacteria were released by lysing the macrophages with 0.05% SDS. The suspensions obtained from the lysed macrophages were immediately diluted in Middlebrook 7H9 and plated on Middlebrook 7H10 to determine viable counts. During the experiments, the macrophage viability was evaluated by staining with Trypan blue.

## Results

### Metal-dependent growth of ESX-3-depleted *M. tuberculosis*

In a previous work we constructed an *M. tuberculosis* conditional mutant (TB79) in which the expression of the *esx-3* gene cluster is repressed by ATc and showed that its growth in Middlebrook 7H9 stopped after few generations upon *esx-3* repression [26].

**Figure 1:**
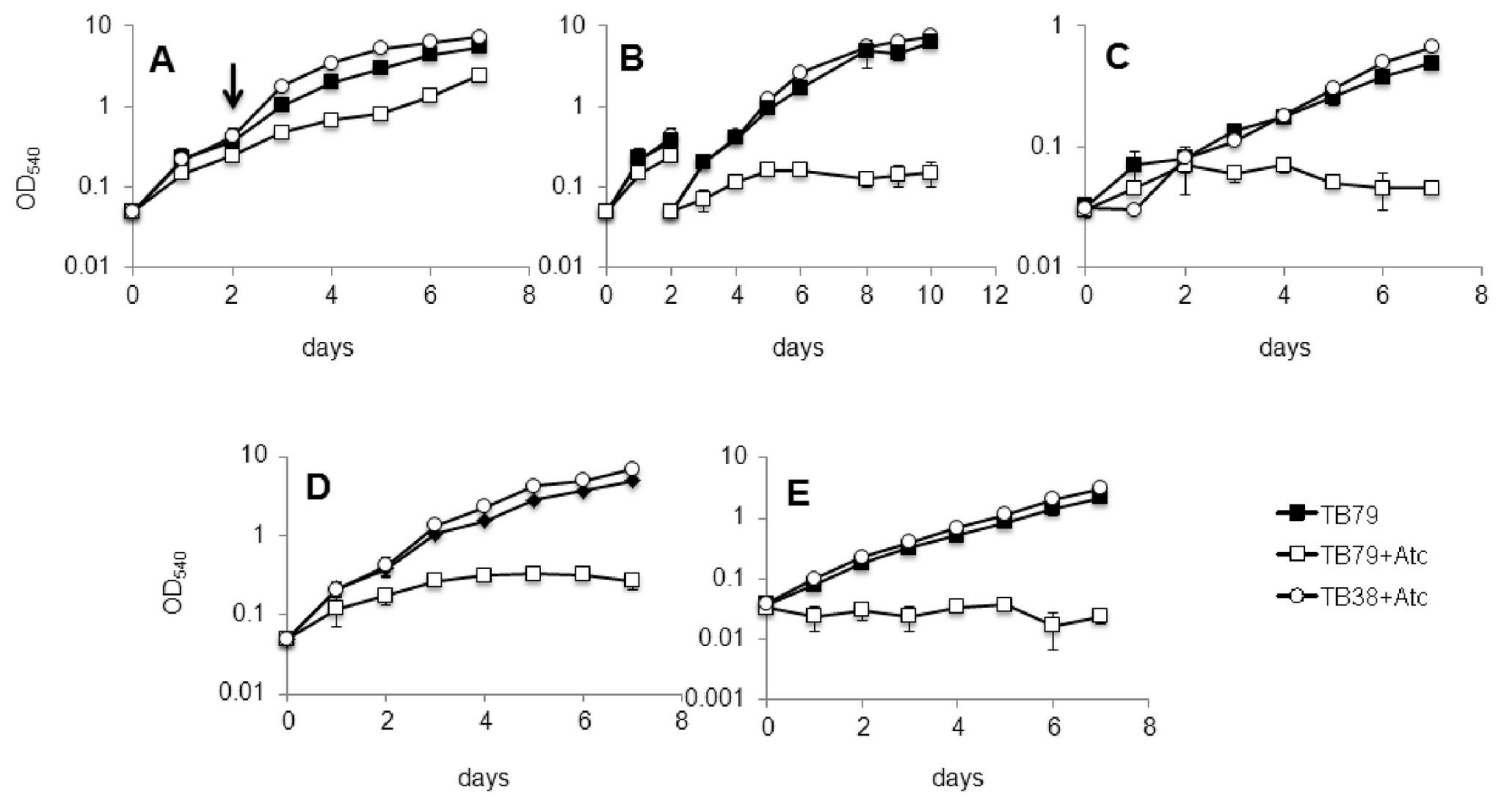
Growth of ESX-3 depleted *M. tuberculosis*. Strains were grown in rolling bottles with or without ATc 200 ng/ml in A) CMM (50μM FeCl_3_ and 3,67 μM ZnSO_4_) first dilution; B) CMM second dilution; C) LIMM (2μM FeCl_3_ and 3,67μM ZnSO_4_) first dilution; D) LZMM (50μM FeCl_3_ and no ZnSO_4_) first dilution; E) Middlebrook 7H9 (^≈^150μM ferric ammonium citrate and ^≈^3,67μM ZnSO_4_) first dilution. The arrow indicates the time point at which the second dilution was performed. Growth of the parental strain in all tested conditions was the same with or without ATc: only growth curve of cultures with ATc are shown. Values represent the average of three independent experiments with relative standard errors.

In *M. tuberculosis* the *esx-3* gene cluster is regulated by the iron dependent repressor IdeR and by the zinc uptake regulator Zur [27,47], suggesting its involvement in the adaptive response to the availability of these two metals. To challenge this hypothesis the *esx-3* conditional mutant (TB79) and its parental strain (TB38) were cultured in defined MM with different concentrations of iron or zinc. In our previous work we found that to detect the TB79 growth phenotype in Middlebrook 7H9, liquid cultures had to be pre-grown in 7H9 in the presence of ATc before being diluted 1:10 in fresh medium containing ATc (Figure 1 E). Applying the same protocol, we found that the conditional mutant TB79 cultured in low iron (LIMM) and low zinc (LZMM) with ATc, stopped dividing after the first dilution (Figure 1 C-D), while in complete MM (CMM) plus ATc its growth was arrested only after a second dilution (Figure 1 A-B). These results indicate that the repression of *esx-3* reduces growth in both low iron and low zinc medium and that sustained repression of this system prevents normal growth even under iron and zinc sufficient conditions. Surprisingly, the growth defect experienced by maintaining ESX-3 repression was more evident in Middlebrook 7H9 than in CMM, despite the presence of an iron concentration three fold higher in Middlebrook 7H9 than in CMM and an equal zinc concentration in the two media.

### Transcriptional profile of ESX-3-depleted *M. tuberculosis*

To better characterize the impact of *esx-3* down-modulation on *M. tuberculosis* physiology, we compared the global transcriptional profiles of the *esx-3* conditional mutant and its parental strain grown in Middlebrook 7H9 supplemented with ATc. Cells were collected after 48 h of exposure to 100 ng/ml of ATc. In these conditions *esx-3* transcription was repressed about 35 fold (Figure S4), while growth and vitality of the mutant were not drastically affected (data not shown). In addition to the genes belonging to the *esx-3* cluster, the expression of 70 genes was significantly altered in the conditional mutant cultured in the presence of ATc. A total of 52 genes were up-regulated and 18 were down-regulated (Table S4). Forty of these genes are regulated by iron and IdeR and encode proteins involved in adaptation to low iron conditions. Up-regulated genes included those encoding the mycobactin synthesis proteins (Mbt) [48], the membrane proteins MmpS4/L4, involved in siderophore export [49], the ABC transporter IrtAB, responsible for Fe^+3^-carboxymycobactin uptake [33] and the SUF [Fe-S] cluster assembly system (Rv1460-Rv1466) [50]. However, the *nuo* operon, encoding the different components of an NADH:ubiquinone oxidoreductase (*rv3145-rv3158*) known to be induced in high iron [47], was down regulated in ESX-3-depleted cells. Eight genes belonged to the Zur regulon, in particular the *rv2055c-rv2058c* operon, encoding ribosomal proteins hypothesized to be involved in the release of zinc from the cytoplasmic stores and the *rv2059-rv2060* operon, encoding a putative zinc transport system [27,51,52] were all up-regulated in the absence of ESX-3. Finally, 22 genes not known to be iron or zinc regulated and encoding proteins of unknown function, proteins involved in stress response as AhpD or proteins containing iron as the NADH dehydrogenase Ndh were either up or down-regulated (Table S4).

Taken together, these results indicate that the conditional mutant grown in Middlebrook 7H9 in presence of ATc (causing the repression of *esx-3*) experienced iron and zinc starvation regardless of the high concentration of these two metals in the medium.

### Expression analysis of iron- and zinc-regulated genes in ESX-3-depleted *M. tuberculosis* during growth in defined metals concentrations

**Figure 2:**
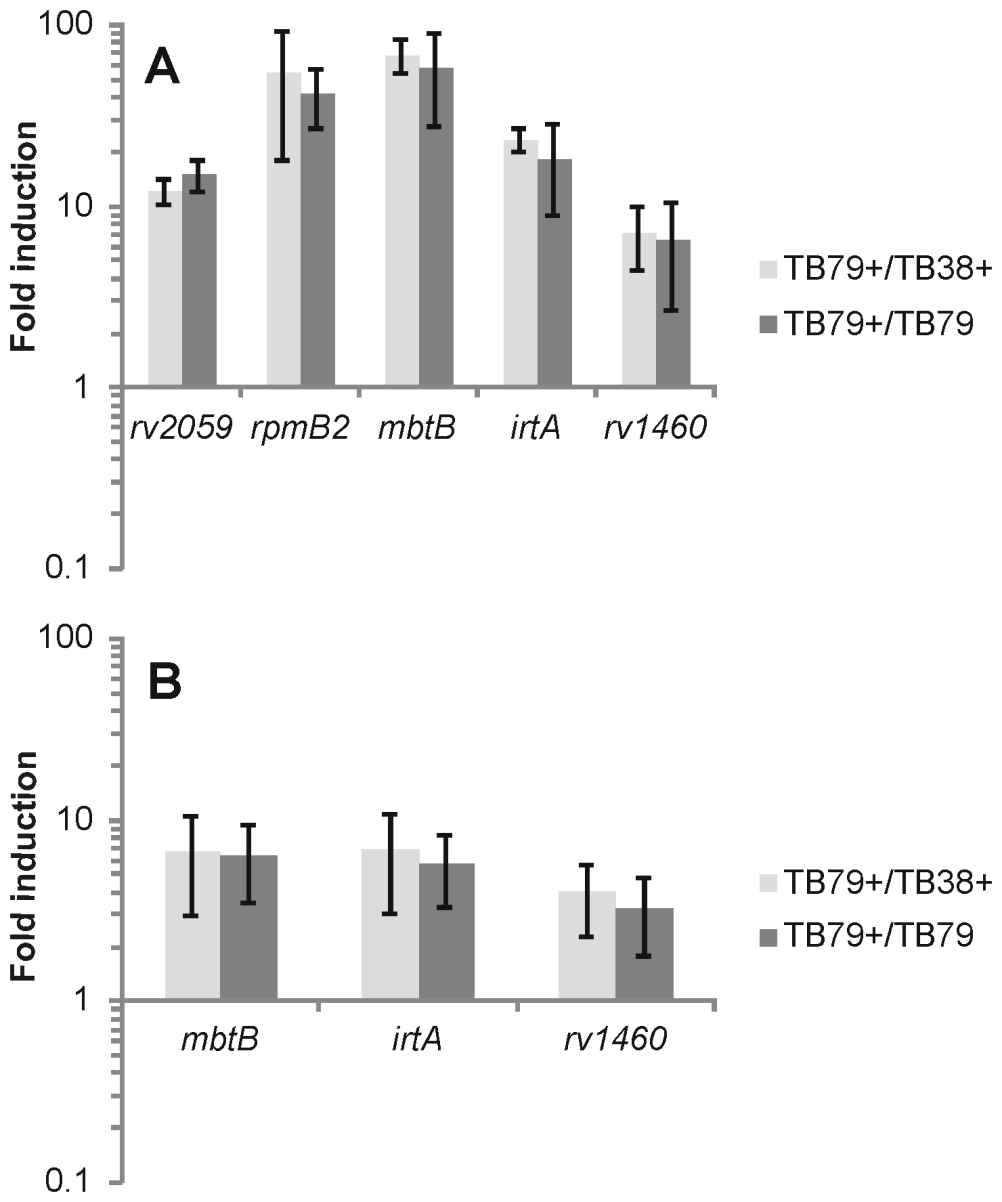
Expression analysis in ESX-3 depleted *M. tuberculosis* in defined medium. Quantitative real-time RT-PCR performed on total RNA extracted from cells grown in CMM (50μM FeCl_3_ and 3,67 μM ZnSO_4_) and in LIMM (2μM FeCl_3_ and 3,67μM ZnSO_4_). The graphs represent the ratio between expression levels (normalized to *sigA* mRNA) in the conditional *esx-3* mutant (TB79) and its parental strain (TB38) grown with or without ATc (+ symbol) in A) CMM and B) LIMM. Values represent the average of three independent experiments with the relative standard errors.

To better characterize the transcriptional response of the ESX-3-deficient *M. tuberculosis* to iron and zinc availability, we examined the expression of three iron-regulated genes (*mbtB*, *irtA*, and *rv1460*) and 2 zinc-regulated genes (*rv2059* and *rpmB2*) by quantitative real-time RT-PCR in bacteria grown in MM with defined concentrations of iron and zinc. Figure 2A shows the ratio of the expression levels of the 5 genes between the conditional mutant and its parental strain grown in CMM in the presence of ATc. The 5 genes were expressed at a higher level in the conditional mutant validating the microarray results and supporting the idea that *esx-3* deficient *M. tuberculosis* experiences iron and zinc limitation despite the abundance of these metals in the medium. Moreover, we also measured the ratio of the expression levels of the same genes in the conditional mutant grown in the presence and absence of ATc. Also in this case, the genes analyzed were expressed at a higher level when *esx-3* was down-regulated by ATc (Figure 2A).

The same experiment was performed in LIMM and again when *esx-3* was down-regulated by ATc the expression of iron-regulated genes increased more than in the parental strain and in the conditional mutant grown without ATc (Figure 2B).

The results of the arrays and quantitative real-time RT-PCR clearly demonstrate that *esx-3* deficiency results in a response that is typical of iron/zinc limitation.

### ESX-3-depleted *M. tuberculosis* contains lower levels of intracellular iron

**Figure 3:**
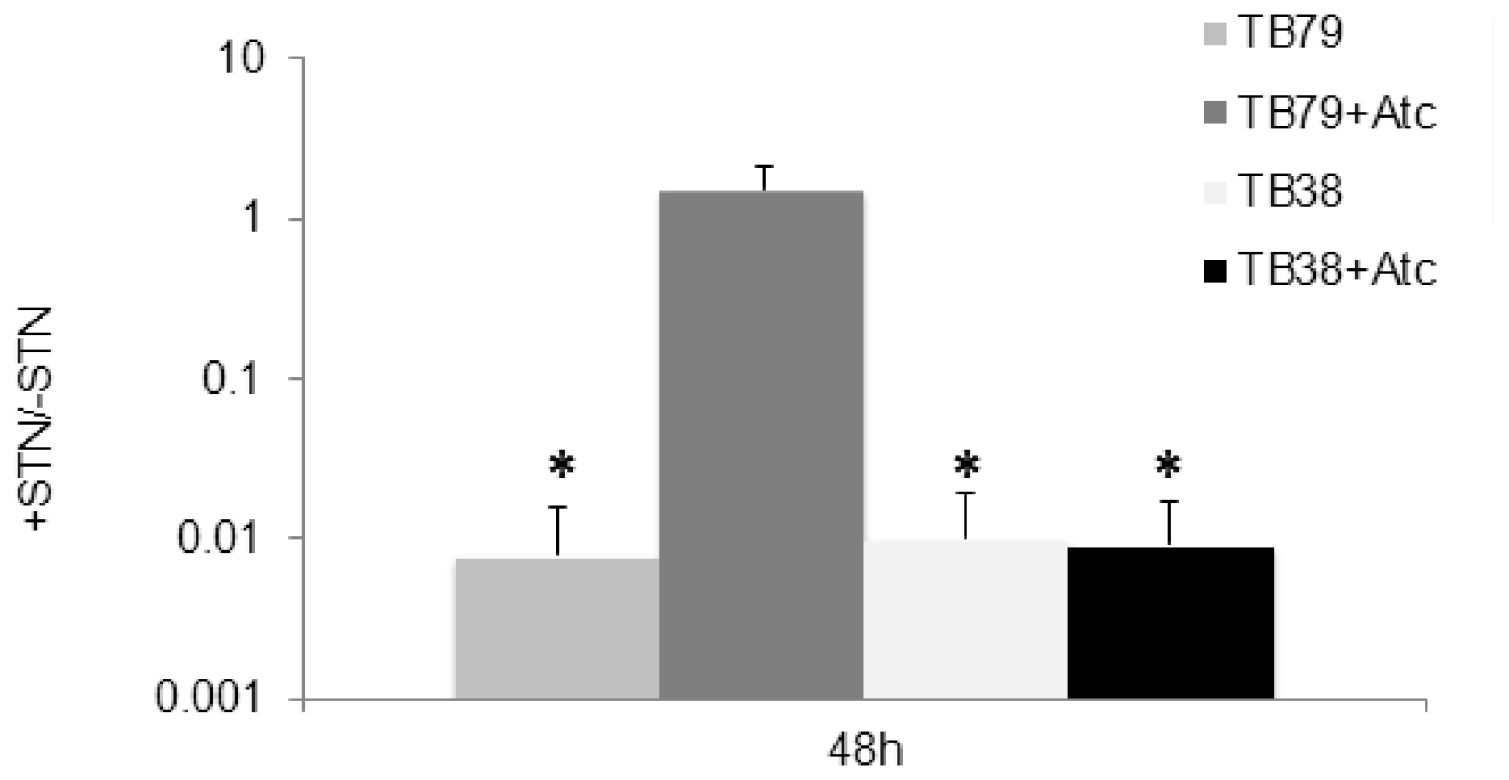
Streptonigrin sensitivity of the *M. tuberculosis* *esx-3* conditional mutant. Cells in exponential phase were treated with 5μg/ml STN (5X MIC) for 48h. The graphs show the survival rate in cultures treated 48 h with STN in respect to untreated cultures (see methods). The values represent the average of three independent experiments with relative standard errors. Asterisks represent statistically significant differences (p<0.05) respect to the conditional mutant (TB79) grown with ATc.

Streptonigrin (STN) can be used as an indirect measure of intracellular iron levels, as low intracellular concentration of this metal reduces the sensitivity to this antibiotic [53]. To further determine whether *M. tuberculosis* is experiencing iron limitation in Middlebrook 7H9 when *esx-3* is repressed, we compared STN sensitivity of the *esx-3* conditional mutant TB79 and its parental strains TB38 in the presence or absence of ATc. As shown in Figure 3 the resistance to STN of the conditional mutant increased about 100 fold in the presence of ATc, suggesting lower intracellular levels of iron in these cells following ESX-3 depletion. 

### Repression of *esx-3* decreases uptake of Fe^+3^-carboxymycobactin

**Figure 4:**
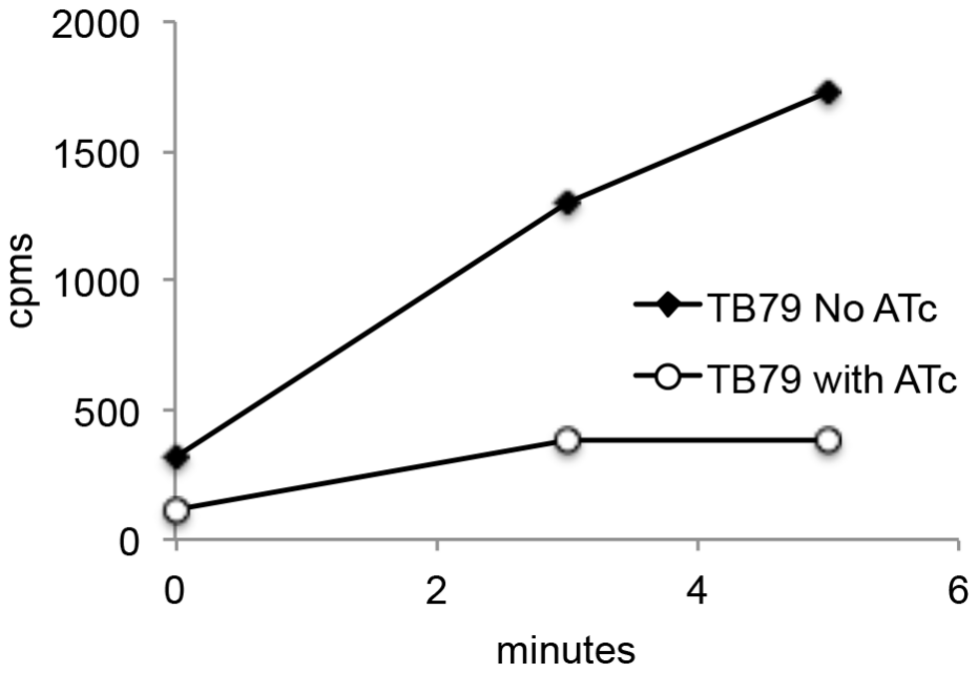
Fe^3^ carboxymycobactin (CM) uptake in ESX-3-depleted *M. tuberculosis*. Incorporation of Fe^55^-CM in TB79 cultured in the presence or absence of ATc was measured as described in materials and methods. The results are the average of duplicates in a representative experiment that was repeated 4 times.

In order to determine whether the iron deficiency phenotype exhibited by TB79 is the result of decreased iron import when *esx-3* is repressed, we measured uptake of ^55^Fe-CM by TB79 and its parental strain in the presence and absence of ATc. No significant difference in the amount of iron taken up by the parental strain in the presence or absence of ATc was observed (data not shown), however, repression of *esx-3* by ATc drastically decreased iron incorporation in TB79 (Figure 4) supporting a role of *esx-3* in iron uptake.

### Iron dependent growth and low intracellular iron concentration of an *M. smegmatis esx-3* null mutant

**Figure 5:**
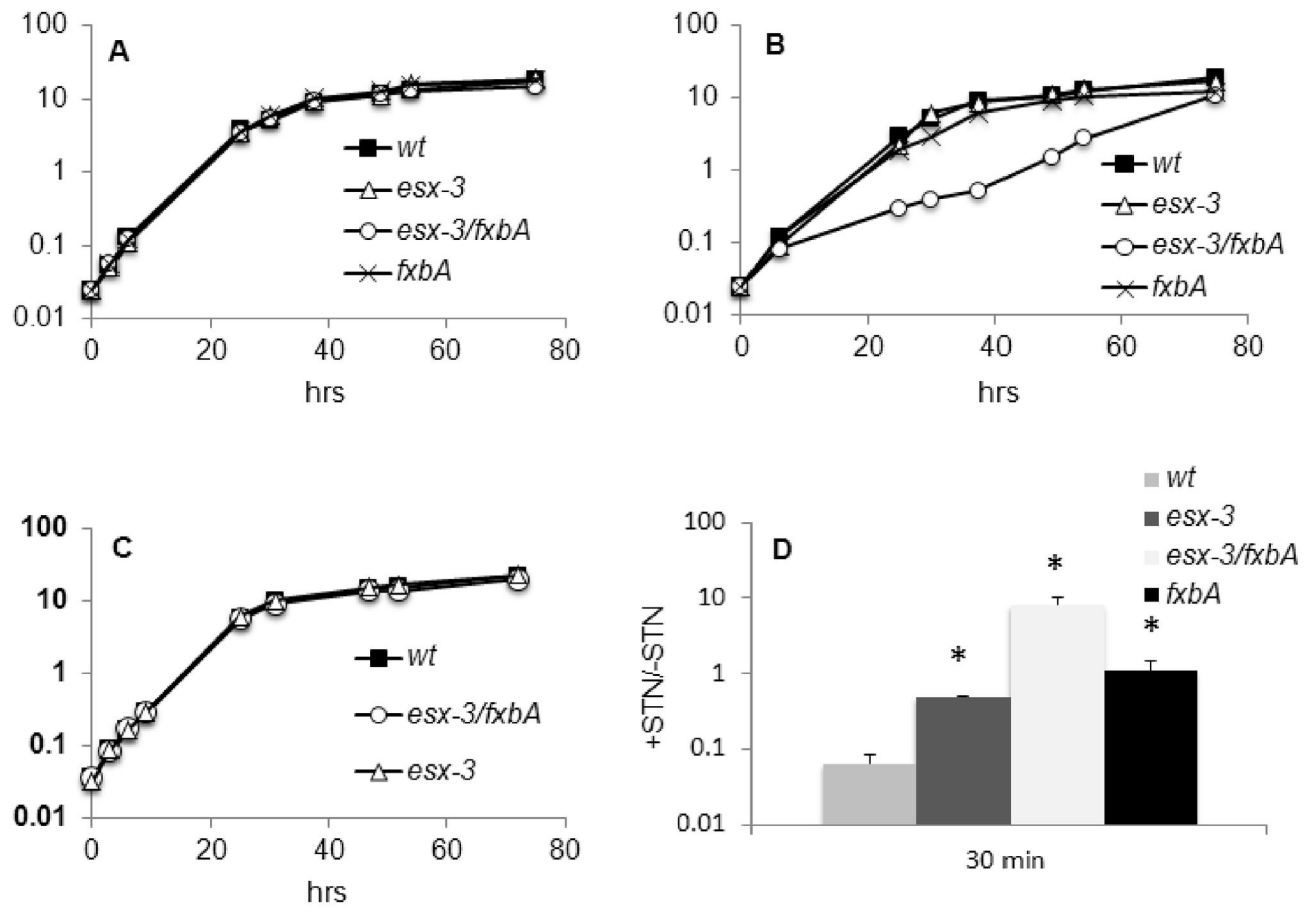
Growth in different metals concentrations and streptonigrin sensitivity of the *M. smegmatis* *esx-3* null mutants. The wild type strain mc^2^155 and its derivatives mutants *esx-3*/*fxbA*, *esx-3*, and *fxbA* were grown in shaking cultures in MM with different iron and zinc concentrations: (A) CMM (50μM FeCl_3_ and 3,67 μM ZnSO_4_); (B) LIMM (2μM FeCl_3_ and 3,67μM ZnSO_4_), (C) LZMM (50μM FeCl_3_ and no ZnSO_4_ added). Cells were pre-grown in CMM. The experiment was repeated three times. Data from one representative experiment are shown. D) Sensitivity to STN: cells in exponential phase were treated with 5μg/ml STN (5X MIC) for 30’; the graphs shows the survival rate in cultures treated 30 min with STN compared with untreated cultures (see methods). The values represent the average of three independent experiments with relative standard errors. Asterisks represent statistically significant differences (p<0.05) respect to the wild type strain.

Since in *M. smegmatis esx-3 gene* cluster is regulated by IdeR and not by Zur, we hypothesized that in this specie this secretion system is only involved in iron uptake. It has been previously shown that in *M. smegmatis esx-3* is essential in iron-limiting conditions when the biosynthetic pathway for exhochelin (the major siderophore in this species) is inactivated [36]. However, the involvement of ESX-3 in zinc uptake has not been investigated. To test our hypothesis, we constructed an *esx-3* and *fxbA* (gene essential for exochelin biosynthesis) null mutants, as well as an *esx-3/fxbA* double mutant in *M. smegmatis* mc^2^155, and analyzed the growth of these mutants in MM with or without iron or zinc (Figure 5). The single mutants did not display any growth defect in all conditions tested, while iron limitation resulted in delayed growth of the double mutant, confirming previous observations [36] (Figure 5B). The growth of the *esx-3-fxbA* double mutant was not affected in medium lacking zinc (Figure 5C), indicating that in *M. smegmatis* ESX-3 is dispensable for growth in low zinc. Sensitivity to STN was also evaluated in these mutants grown in LIMM. All mutants showed an increased resistance to STN compared to their wild type strain: in particular, the double *esx-3/fxbA* mutant was the most resistant and the single *esx-3* mutant was the most sensitive (Figure 5D). 

### Growth of the ESX-3-depleted *M. tuberculosis* and *M. smegmatis* in the presence of heme as iron source

**Figure 6:**
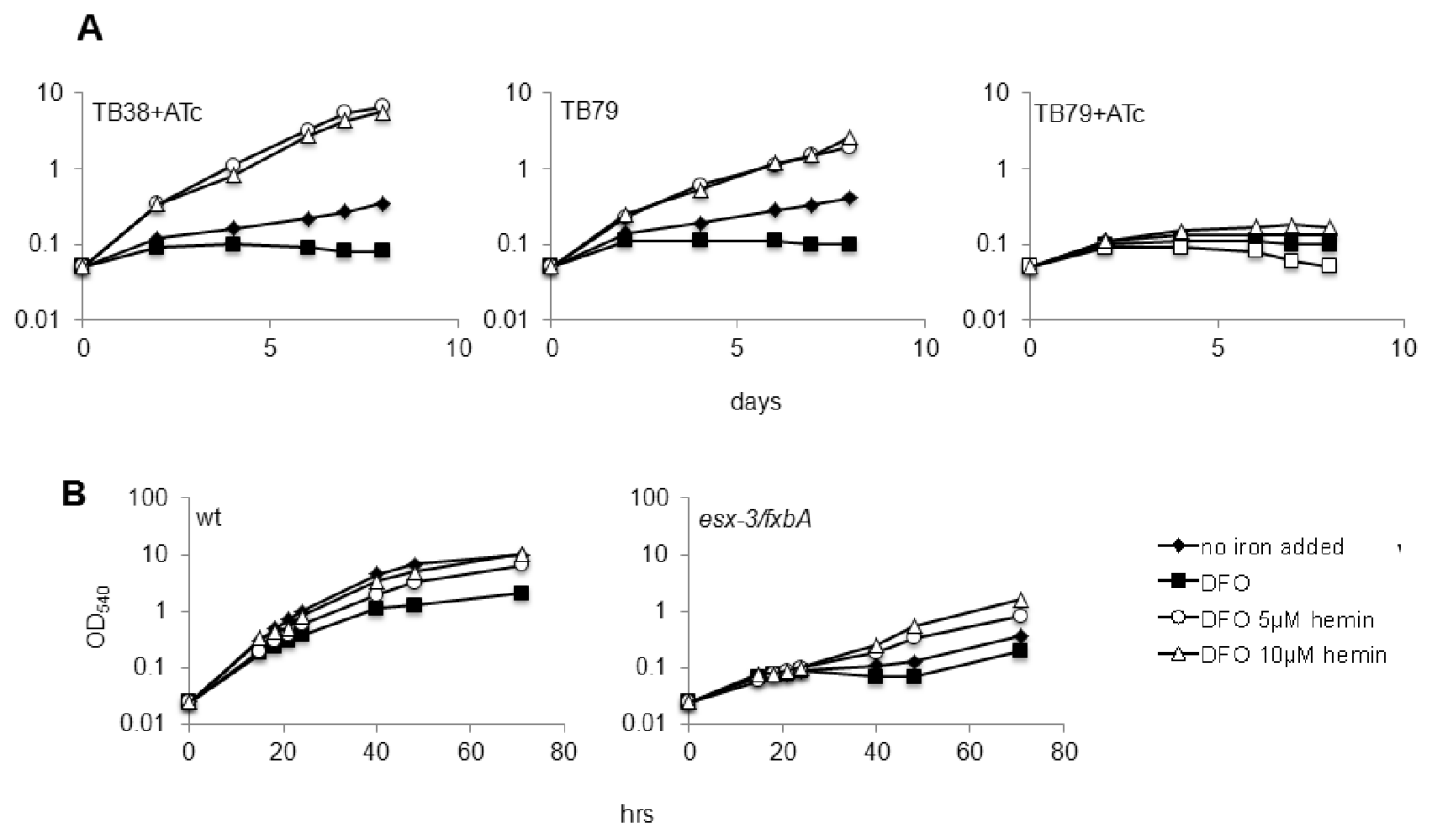
Growth in hemin-supplemented medium. The strains were grown in NIMM (3,67μM ZnSO_4_ and no FeCl_3_) with or without 50 µM DFO and 5 or 10 µM hemin. (A) *M. tuberculosis* *esx-3* conditional mutant (TB79) and its parental strain (TB38) grown with or without 200ng/ml of ATc. The growth of the parental strain in all tested conditions was the same with or without ATc and only growth curve of cultures with ATc are shown. (B) *M. smegmatis* *esx-3*/*fxbA* mutant and its parental wild type strain. Values represent one representative experiment out of three performed.

In addition to siderophores, mycobacteria have systems to acquire iron from the heme prosthetic group [54,55]. To determine whether ESX-3 is necessary for utilization of heme as iron source we grew the *M. tuberculosis esx-3* conditional mutant and the *M. smegmatis esx-3/fxbA* null mutant in MM with hemin as the only iron source. As showed in Figure 6A, the growth of the *M. tuberculosis esx-3* conditional mutant was not rescued by hemin when it was grown in the presence of ATc, while its growth improved in the presence of hemin if ATc was not present. However, hemin was able to clearly enhance the growth of the *M. smegmatis esx-3/fxbA* double mutant (Figure 6B). These results clearly demonstrate that *M. smegmatis* does not require ESX-3 to utilize hemin. The lack of rescue by hemin in *M. tuberculosis* suggests that ESX-3 is necessary for hemin utilization or, alternatively that deficient zinc acquisition prevented normal growth even in the presence of heme.

### ESX-3 depletion doesn’t affect cell wall permeability

It is known that depletion of some components of ESX-1 and ESX-5 cause defects in cell wall permeability [15,56]. This might indicate a common role of T7SS in the regulation of cell wall integrity. To determine whether ESX-3 deficiency causes changes in cell wall permeability, we measured the resistance to antibiotics with different structures as well as sensitivity to SDS in the *M. smegmatis esx-3* mutant. The MICs to ampicillin, rifampicin and Km in Middlebrook 7H9, as well as sensitivity to SDS were the same in the *esx-3* null mutant and in its parental strain (Figures S5 and S6). Since ESX-3 is induced in low iron we evaluated the MICs to these antibiotics also in this condition; however, even in these conditions we could not observe any difference between the two strains (data not shown). The same experiments were performed in the *M. tuberculosis esx-3* conditional mutant grown with or without ATc. Even in this case we could not observe any difference in MIC or SDS sensitivity due to the absence of ESX-3 (data not shown). Thus, these results suggest that no drastic effects in cell wall permeability occur as a result of ESX-3 depletion in *M. smegmatis* or in *M. tuberculosis*.

### ESX-3 depletion prevents *M. tuberculosis* replication in THP-1-derived macrophages

**Figure 7:**
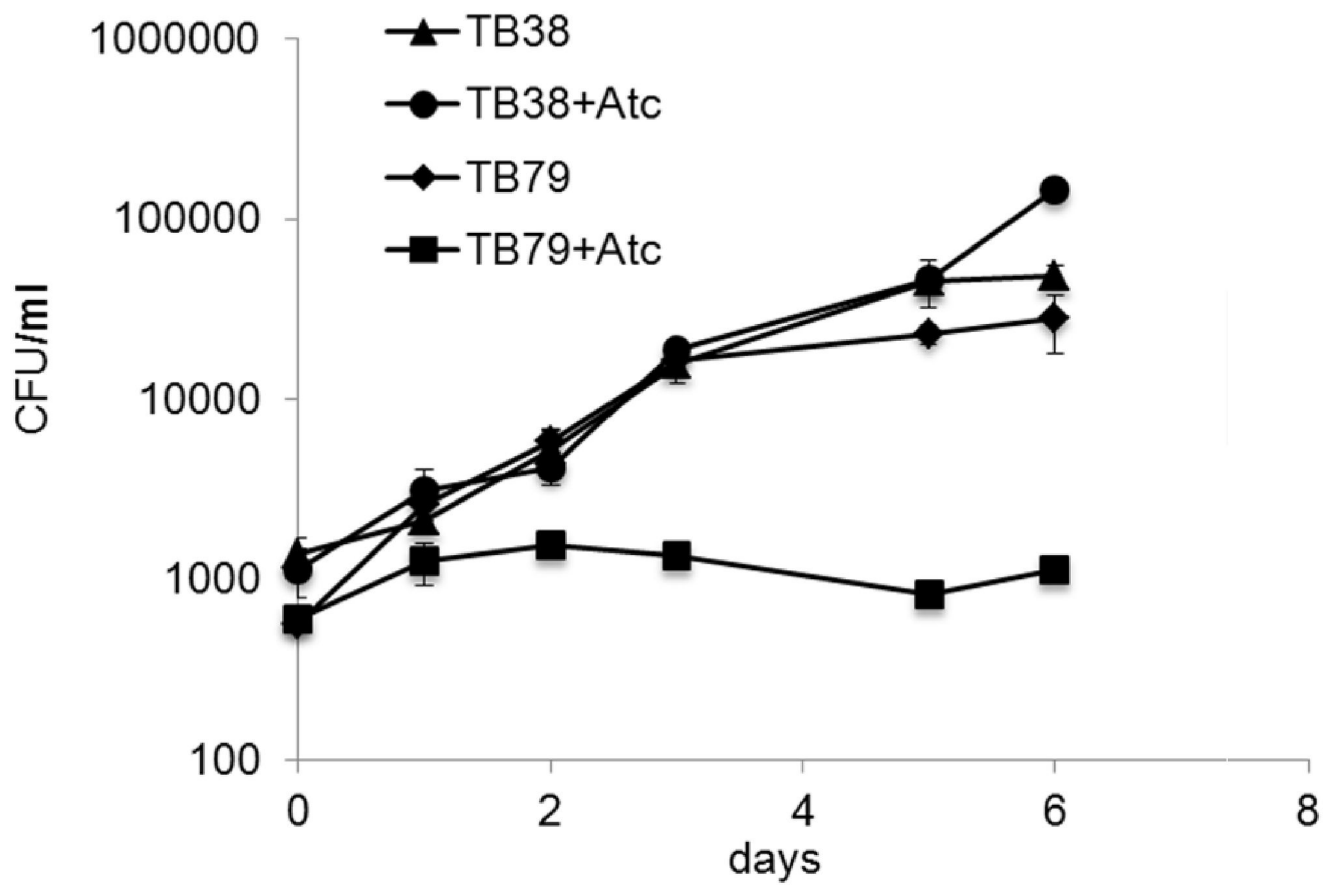
Infection of THP1-derived macrophages. THP1-derived macrophages were infected with conditional mutant (TB79) and parental strain (TB38) at an MOI of 1:20 in presence or absence of 200 ng/ml of ATc. The growth curve was measured as CFU/ml after release of intracellular bacteria in 100 µl of 0.05% SDS . The values represent the average of two independent experiments with relative standard error.

To determine whether ESX-3 is essential for intracellular replication of *M. tuberculosis*, we infected THP-1-derived macrophages with the *esx-3* conditional mutant in the presence or absence of ATc. As shown in Figure 7 the mutant was not able to replicate in these macrophages when ATc was added to the culture medium indicating that ESX-3 is necessary for intracellular growth of *M. tuberculosis*.

## Discussion

The role of ESX-3 in *M. smegmatis* and *M. tuberculosis* has been examined previously [26,36]. In both species it was clearly shown to be necessary for adaptation to iron limitation, however, differences in its physiological role in the two species were evident: i) while in *M. smegmatis esx-3* expression is repressed by IdeR, in *M. tuberculosis* is repressed by both IdeR and Zur; ii) in *M. tuberculosis* ESX-3 is essential under standard culture conditions, while in *M. smegmatis* it becomes essential only if the biosynthetic pathway for its major siderophore (exochelin) is disrupted by *fxbA* deletion, and bacteria are grown in low iron conditions; iii) finally, the *esx-3* gene cluster of *M. tuberculosis* is not able to complement the phenotypes associated with *M. smegmatis esx-3* deletion [36,57].

In this study we further characterized the role of ESX-3 in these two species comparing the behavior of an *M. smegmatis esx-3*/*fxbA* null mutant to that of a conditional *esx-3* mutant of *M. tuberculosis* grown in the same conditions.

The growth defect of *M. tuberculosis* lacking ESX-3 in iron deficient conditions correlated with reduced uptake of Fe^3+^-carboxymycobactin (Figure 4) indicating participation of ESX-3 components in iron import, something that has been postulated but not demonstrated [36]. How ESX-3 contributes to iron uptake is an open question. Components of the ESX-3 system may facilitate transit of Fe^3+^-carboxymycobactin through the cell wall to reach IrtAB in the plasma membrane, a secreted ESX-3 substrate may be required for proper iron-siderophore uptake or ESX-3 may create the right cell surface environment for iron uptake. Those are all possibilities to be tested. Interestingly, ESX-3 deficiency also decreased *M. tuberculosis* but not *M. smegmatis* capacity to utilize heme as iron source, which raises the question of whether ESX-3 is also directly involved in heme uptake or the iron-siderophore and heme utilization pathways are somehow connected in *M. tuberculosis*. 

In iron sufficient environments, cells still require iron. Low affinity iron acquisition pathways can satisfy that need. Indeed, in high-iron conditions *M. smegmatis* can acquire iron through Msp porins [58]. However, low affinity iron uptake systems have not been characterized in *M. tuberculosis*. Differences in low affinity iron uptake pathways may explain the divergent requirement for ESX-3 observed between *M. tuberculosis* and *M. smegmatis* in high iron medium. 

While in *M. smegmatis* ESX-3 is completely dispensable in Middlebrook 7H9 (data not shown) and in CMM (Figure 5A), *M. tuberculosis*’ ESX-3 is essential in those conditions (Figure 1 BE). It is possible that in *M. tuberculosis* ESX-3 is needed for iron and zinc uptake even in medium rich in these metals. The altered expression of iron regulated genes in ESX-3 depleted *M. tuberculosis* growing in Middlebrook 7H9 or in CMM as shown by DNA microarrays analyses and quantitative real-time RT-PCR (Table S4 and Figure 2) supports this hypothesis and indicates that repression by IdeR and Zur [26,47] may not be absolute and low levels of *esx-3* expression and siderophore uptake sustain growth of *M. tuberculosis* in high iron and zinc medium. This interpretation is supported by the deficient growth of various siderophore synthesis-mutants in high iron conditions [59,60] and is consistent with the decreased sensitivity to streptonigrin observed when TB79 was cultured in high iron in the presence of ATc. It also agrees with the small amounts of *rv0282* RNA that are detected by quantitative real-time RT-PCR in *M. tuberculosis* growing in Middlebrook 7H9 and CMM (data not shown).

We showed that the growth defect experienced by following ESX-3 depletion was more evident in Middlebrook 7H9 than in CMM (Figure 1 B-E). A possible explanations for this surprising finding is that iron and/or zinc bioavailability in Middlebrook 7H9 is lower than in CMM, or that ESX-3 is involved in some other, still unrecognized process beyond iron and zinc metabolism.

Although, zinc uptake was not measured, the growth defect in LZMM and the up-regulation of genes belonging to the *zur* operon in high zinc medium in ESX-3- depleted *M. tuberculosis* suggest that ESX-3 can be involved in zinc acquisition in *M. tuberculosis*. Zinc uptake in *M. tuberculosis* has not been characterized, but some hypothesis can be drawn analyzing the members of the Zur regulon [27]. Beyond genes encoding ESX-3 and proteins hypothesized to be involved in the mobilization of intracellular zinc storage, Zur regulates *rv0106*, a gene encoding a protein similar to the *Bacillus subtilis* putative zinc low-affinity transporter YciC [61], and two overlapping genes (*rv2059-rv2060*) encoding an incomplete putative ABC transporter. Interestingly, the final part of *rv2059* totally overlaps *rv2060*, even if using a different reading frame due to a deletion leading to the fusion of the terminal part of *rv2059* with a sequence internal to *rv2060*. This deletion occurs also in *Mycobacterium bovis*, but not in other mycobacteria as *Mycobacterium leprae*, *Mycobacterium avium* or *Mycobacterium marinum*, where the structure of the ABC transporter system is intact [27]. Whether Rv2059 and Rv2060 are still functional is unknown, but seems unlikely given the large extent of the lesion. We can hypothesize that while the transport of zinc across the plasma membrane is due to Rv0106 in *M. tuberculosis* and *M. bovis*, and to the complete ABC transporter in the other mycobacteria, ESX-3 might facilitate the access of zinc to the periplasmic space, by a still unknown mechanism. 

It has been recently shown that EsxH, one of the members of the main ESX-3 secreted target (EsxG/EsxH heterodimer) contains a zinc-binding site. Coordination of zinc from this protein was proposed to modify the structure of a cleft on the surface of the EsxG/EsxH heterodimer probably involved in the interaction with other binding partners [62]. Whether EsxH binding to zinc reflects a direct role in zinc scavenging and acquisition or a zinc-dependent regulation of the interaction of the EsxG/EsxH heterodimer with other partner proteins, is still unknown. However, the fact that the zinc binding site is only conserved in obligate pathogens as *M. tuberculosis*, *M. bovis* and *M. leprae*, but not in environmental, opportunistic pathogens as *M. marinum*, *M. ulcerans*, or *M. smegmatis*, strongly suggests that it might be involved in the survival during infection [62].

The evidence that ESX-3 is necessary in the adaptation to low iron condition but not in low zinc condition in *M. smegmatis* while is necessary in the adaptation to both conditions in *M. tuberculosis* indicates that the different regulation of the expression of *esx-3* in the two species reflects a different role of this secretion system between them. Interestingly, the Zur binding box upstream of *esx-3*, beyond in *M. tuberculosis* is present in several slow growing mycobacteria, as *M. bovis*, *M. ulcerans*, *M. leprae*, *M. marinum*, and *Mycobacterium simiae*, but is absent in other rapid growers beyond *M. smegmatis* as *Mycobacterium gilvum* and *Mycobacterium abscessus* despite the presence of Zur in this bacteria (data not shown). These findings suggest that zinc-related functions of ESX-3 were acquired by slow growers after their phylogenetic separation from rapid growers.

Finally, in agreement with the data previously obtained in *M. bovis* BCG [36], we showed that ESX-3 is required for the growth of *M. tuberculosis* inside THP-1-derived human macrophages.

In conclusion, our data show that ESX-3 is involved in the uptake of iron and possibly zinc. These two metals are chemically and structurally different and it seems improbable that the same channel or mechanism could be responsible for their import inside the cell. A possibility is that one of the ESX-3 secreted substrates may be necessary for optimal import of these metals. The form in which zinc is acquired is unknown, the possibility that it might be acquired as zinc-loaded EsxH is an intriguing possibility that needs to be tested. Moreover, although, we did not detect drastic changes in cell wall permeability based on sensitivity to antibiotics and SDS is still possible that local changes on the cell wall in the absence of ESX-3 affect transport of diverse molecules.

Interestingly, intraphagosomal iron and zinc concentrations change during infection [63,64]. While iron depletion is a common mechanism of nutritional immunity adopted from the host cell to defeat bacteria [65,66], zinc poisoning has recently emerged as a possible weapon against intracellular *M. tuberculosis* [67,68]. After macrophage activation, iron concentration in the *M. tuberculosis*-containing phagosomes decreases [63] with the aim to starve the bacteria for this essential metal, while that of zinc increases with the aim to intoxicate the bacteria. This raises the question of how metal concentrations regulate ESX-3 *in vivo* since one promoter will be down-regulated by high zinc (Zur-dependent), while the other will be up-regulated in response to low iron (IdeR-dependent). It is possible that the two metal-dependent promoters together with other regulatory elements, as the riboswitch element in the 5’ UTR *esx-3* operon transcript recently reported by Arnvig and coll [69], are needed to guarantee a very sensitive fine-tuning of ESX-3 expression to allow the bacteria to acquire the proper amount of iron and avoiding an excessive uptake of zinc which might lead to their intoxication. We propose that ESX-3 has a crucial role in the maintenance of iron and zinc homeostasis during infection, acting as a defense against nutritional immunity.

## Supporting Information

Figure S1 **Repression of *esx-3* in the *M. tuberculosis* conditional mutant TB79 grown in Middlebrook 7H9 with 100ng/ml of ATc.** The cells were grown in Middlebrook 7H9 with or without 100ng/ml of ATc in rolling cultures and total RNA was extracted after 48h. The amount of RNA specific for *rv0282* (the first gene of *esx-3* gene cluster), was determined by quantitative real-time RT-PCR and normalized with the amount of sigA-specific RNA. The primers used are listed in Table S3. The primers amplify a region located after the homologous regions used to construct the TB79 conditional mutant (Serafini, 2009, J Bacteriol 191: 6340).
(TIF)

Figure S2 **Construction of the suicide vector pAGN27.** Representation of the *esx-3* *locus* and the primers used for amplification of the two 1Kb *esx-3* flanking regions used for homologous recombination. The two fragments were stitched and cloned in p2NIL (Parish and Stoker, 2000, Microbiology, 146:1969) to obtain pAGN25. The *lacZ/sacB/hyg* cassette from pGOAL19 (Parish and Stoker, 2000 Microbiology, 146:1969) was then inserted in PacI restriction site in pAGN25 to obtain the final suicide vector pAGN27.
(TIF)

Figure S3 **Unmarked deletion of the *esx-3locus* in *M. smegmatis*.** A) Schematic representation of the two possible recombination events leading the integration of pAGN27 in the *esx-3* *locus*. B) Two possible structures of the *esx-3* *locus* after integration of pAGN27 and schematic representation of the second recombination event leading to the deletion of the 14.5 kb containing 9 of 11 genes of the *esx-3* *locus*. C) Schematic representation of the *esx*-*3* locus structure after deletion. The primers used to verify by PCR the homologous recombination are indicated (Table S2).
(TIF)

Figure S4 **Unmarked deletion of *fxbA* in *M. smegmatis*.** Schematic representation of the strategy used to obtain the in-frame deletion of *fxbA*: this gene was replaced by double cross-over with an *hyg*-*dif* cassette. Then, homologous recombination between the *dif* sequences allowed the excision of the higromycin resistance gene. The primers used to amplify the regions used for homologous recombination and the primers used to verify the integration and the following deletion are indicated (Table S2).
(TIF)

Figure S5 **Determination of the MIC to different antibiotics in the *M. smegmatisesx-3* null mutant.** The y-axes reports the normalized fluorescence signal from Alamar blue dye, while the x-axes indicates antibiotic concentrations. The fluorescent signal was normalized respect to the fluorescence obtained from cultures not treated with drugs. The trend-lines are shown.
(TIF)

Figure S6 **Survival of the *M. smegmatisesx-3* null mutant after exposure to 0.1% SDS.** The experiment, plated in triplicate, was repeated twice using independent mycobacterial cultures. Values represent the average and the error standard obtained for each point in one representative experiment.
(TIF)

Table S1 **List of the strains and plasmids used and constructed in this study.**
(PDF)

Table S2 **List of primers used in this study for mutants construction.**
(PDF)

Table S3 **List of the primers used in for real time RT-PCR.**
(PDF)

Table S4 **Differentially regulated genes in ESX-3-depleted *M. tuberculosis.***
(PDF)
