# Correction: The ESX-3 Secretion System Is Necessary for Iron and Zinc Homeostasis in *Mycobacterium tuberculosis*

**DOI:** 10.1371/annotation/56401af3-74aa-4c4f-882c-06481b29aa94

**Published:** 2014-01-06

**Authors:** Agnese Serafini, Davide Pisu, Giorgio Palù, G. Marcela Rodriguez, Riccardo Manganelli

Supplementary Figures S1 and S4 were incorrectly switched. The image currently appearing as Figure S1 belongs with the title and legend for Figure S4, and the image currently appearing as Figure S4 belongs with the title and legend for Figure S1. The titles and legends themselves are in correct order. 

