# Supplementary figures and images for: The ESX-3 Secretion System Is Necessary for Iron and Zinc Homeostasis in Mycobacterium tuberculosis
Source: PLoS One. 2013 Oct 14;8(10):e78351. doi: 10.1371/journal.pone.0078351 (PMC3796483; doi:10.1371/journal.pone.0078351)

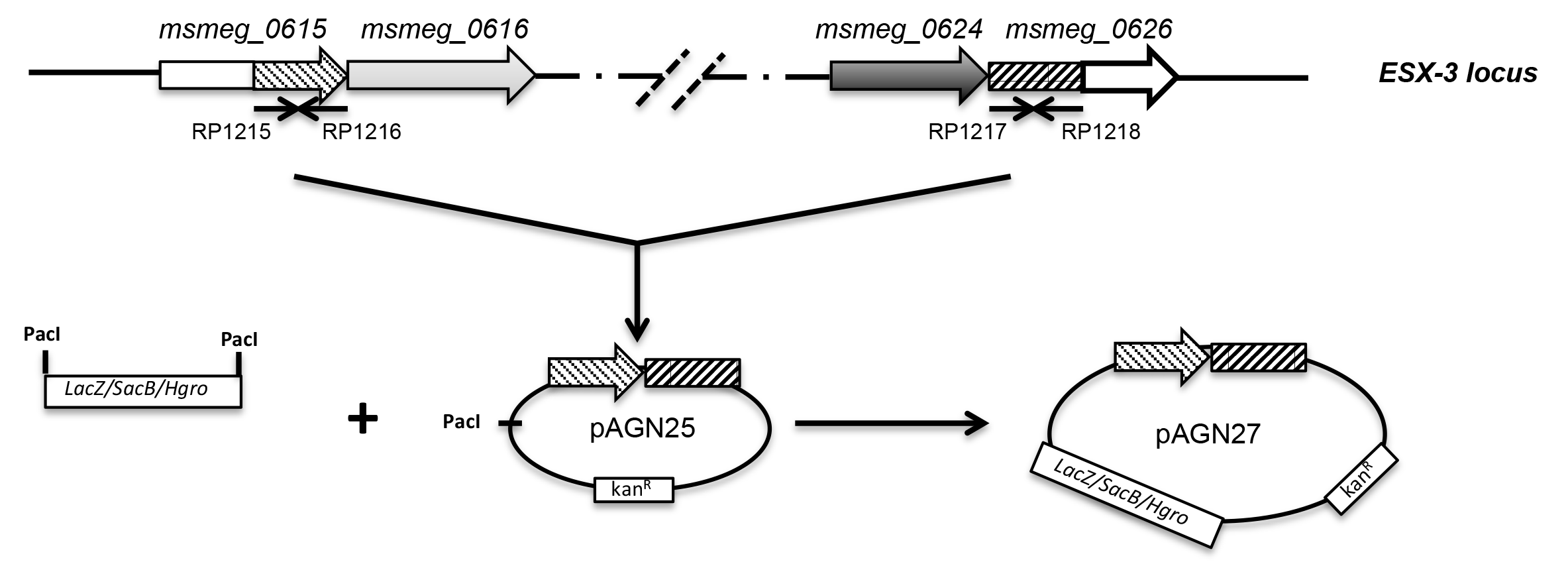

Supplement: Figure S1 — Repression of esx-3 in the M. tuberculosis conditional mutant TB79 grown in Middlebrook 7H9 with 100ng/ml of ATc. The cells were grown in Middlebrook 7H9 with or without 100ng/ml of ATc in rolling cultures and total RNA was extracted after 48h. The amount of RNA specific for rv0282 (the first gene of esx-3 gene cluster), was determined by quantitative real-time RT-PCR and normalized with the amount of sigA-specific RNA. The primers used are listed in Table S3. The primers amplify a region located after the homologous regions used to construct the TB79 conditional mutant (Serafini, 2009, J Bacteriol 191: 6340). (TIF) [file pone.0078351.s001.tif]

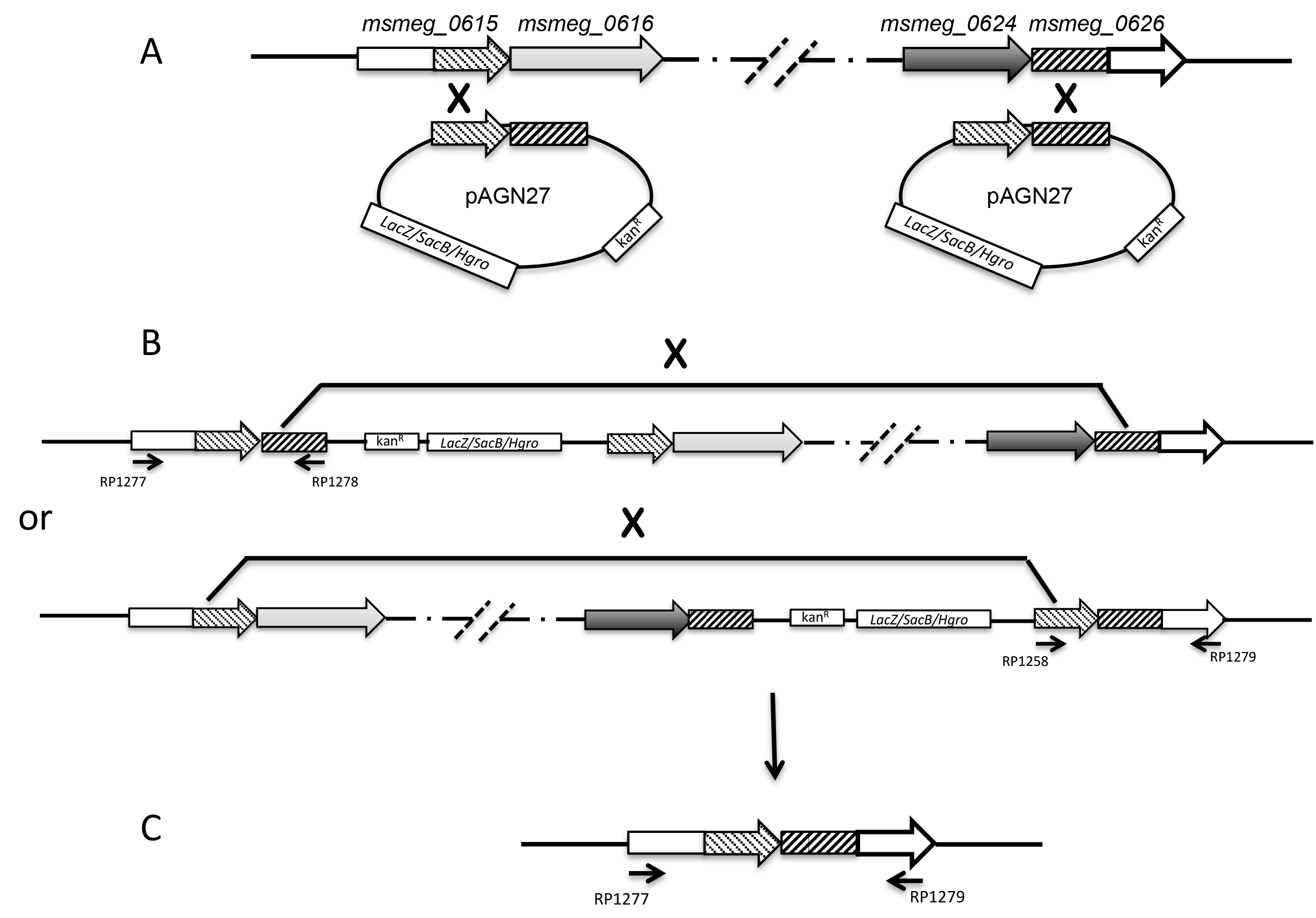

Supplement: Figure S2 — Construction of the suicide vector pAGN27. Representation of the esx-3 locus and the primers used for amplification of the two 1Kb esx-3 flanking regions used for homologous recombination. The two fragments were stitched and cloned in p2NIL (Parish and Stoker, 2000, Microbiology, 146:1969) to obtain pAGN25. The lacZ/sacB/hyg cassette from pGOAL19 (Parish and Stoker, 2000 Microbiology, 146:1969) was then inserted in PacI restriction site in pAGN25 to obtain the final suicide vector pAGN27. (TIF) [file pone.0078351.s002.tif]

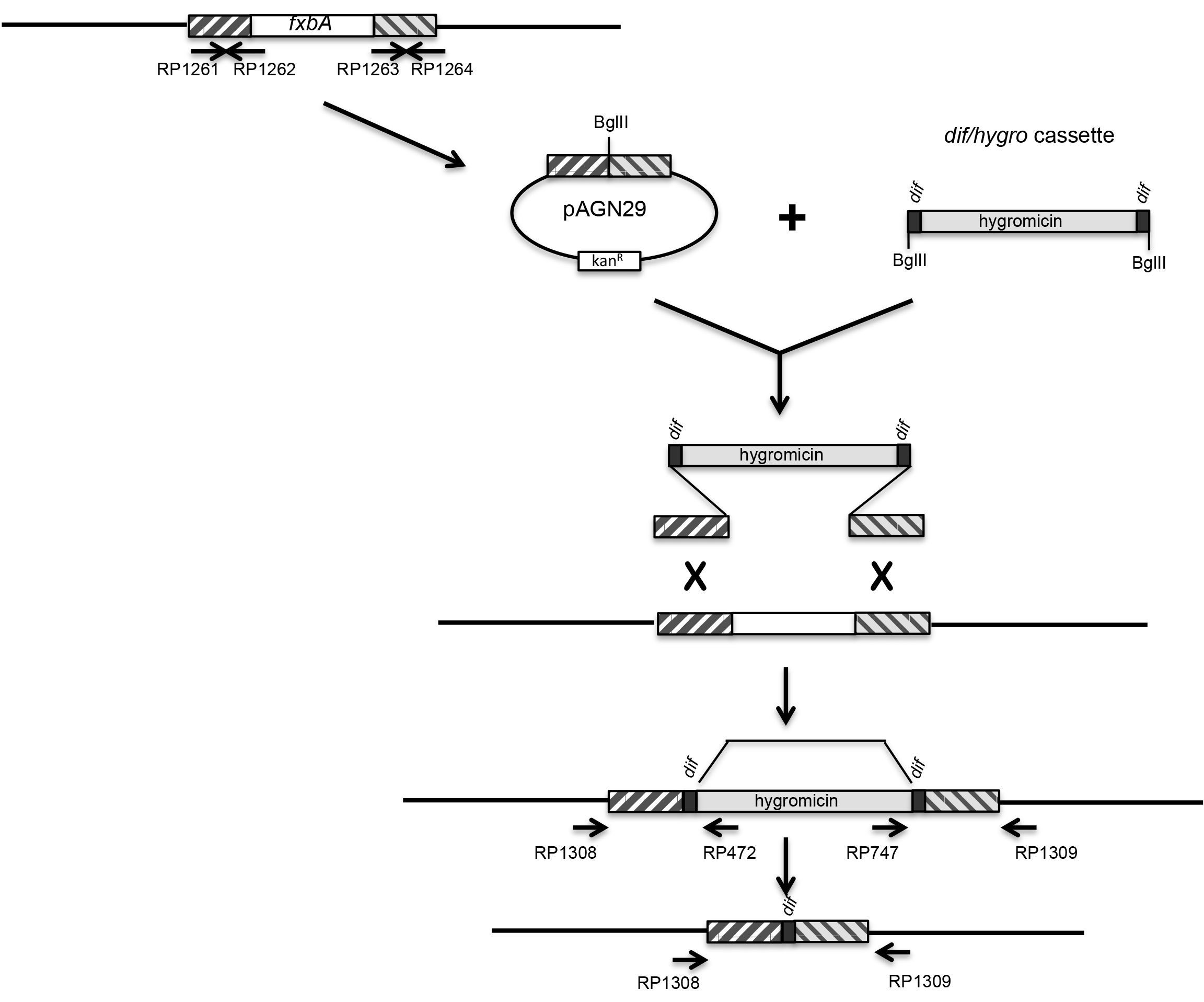

Supplement: Figure S3 — Unmarked deletion of the esx-3locus in M. smegmatis. A) Schematic representation of the two possible recombination events leading the integration of pAGN27 in the esx-3 locus. B) Two possible structures of the esx-3 locus after integration of pAGN27 and schematic representation of the second recombination event leading to the deletion of the 14.5 kb containing 9 of 11 genes of the esx-3 locus. C) Schematic representation of the esx-3 locus structure after deletion. The primers used to verify by PCR the homologous recombination are indicated (Table S2). (TIF) [file pone.0078351.s003.tif]

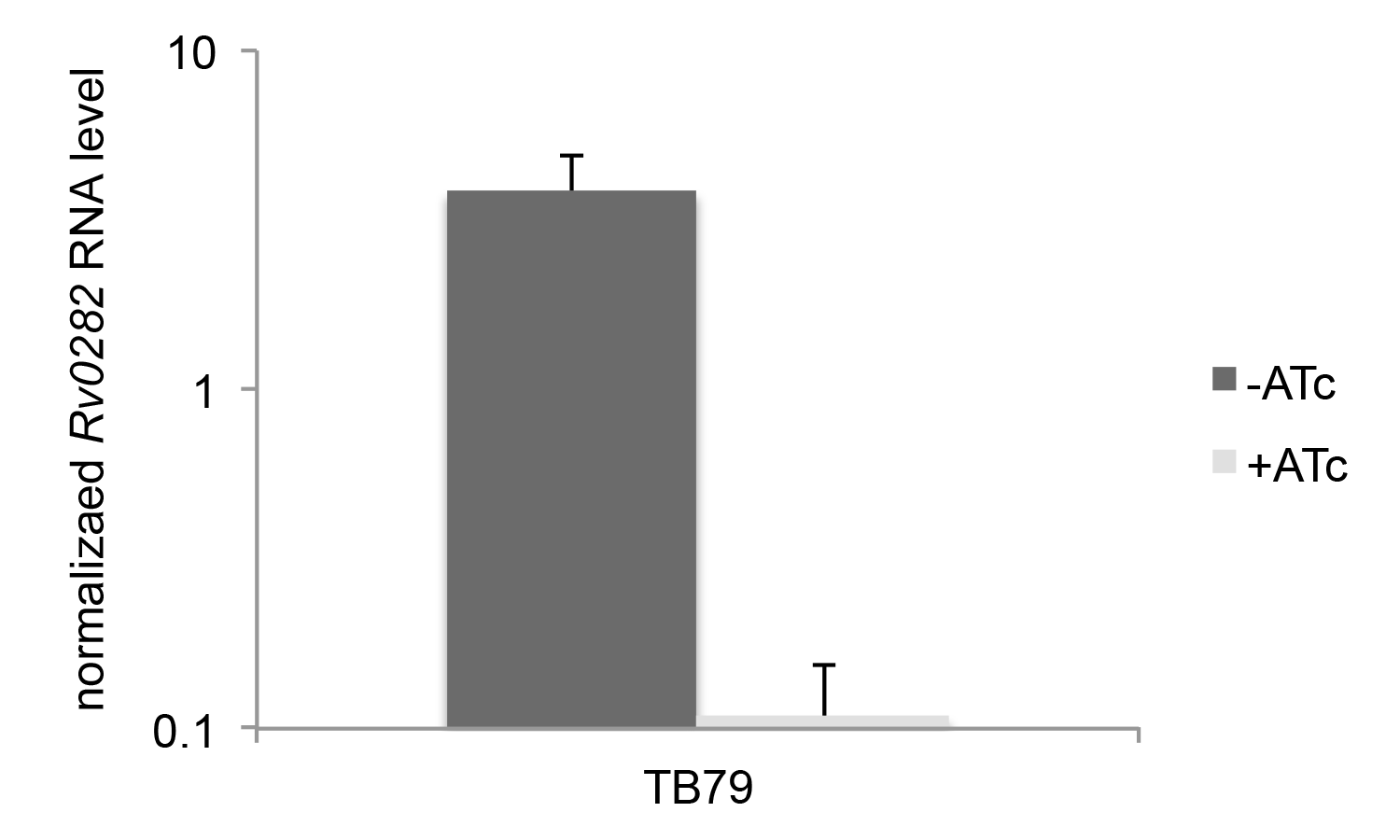

Supplement: Figure S4 — Unmarked deletion of fxbA in M. smegmatis. Schematic representation of the strategy used to obtain the in-frame deletion of fxbA: this gene was replaced by double cross-over with an hyg-dif cassette. Then, homologous recombination between the dif sequences allowed the excision of the higromycin resistance gene. The primers used to amplify the regions used for homologous recombination and the primers used to verify the integration and the following deletion are indicated (Table S2). (TIF) [file pone.0078351.s004.tif]

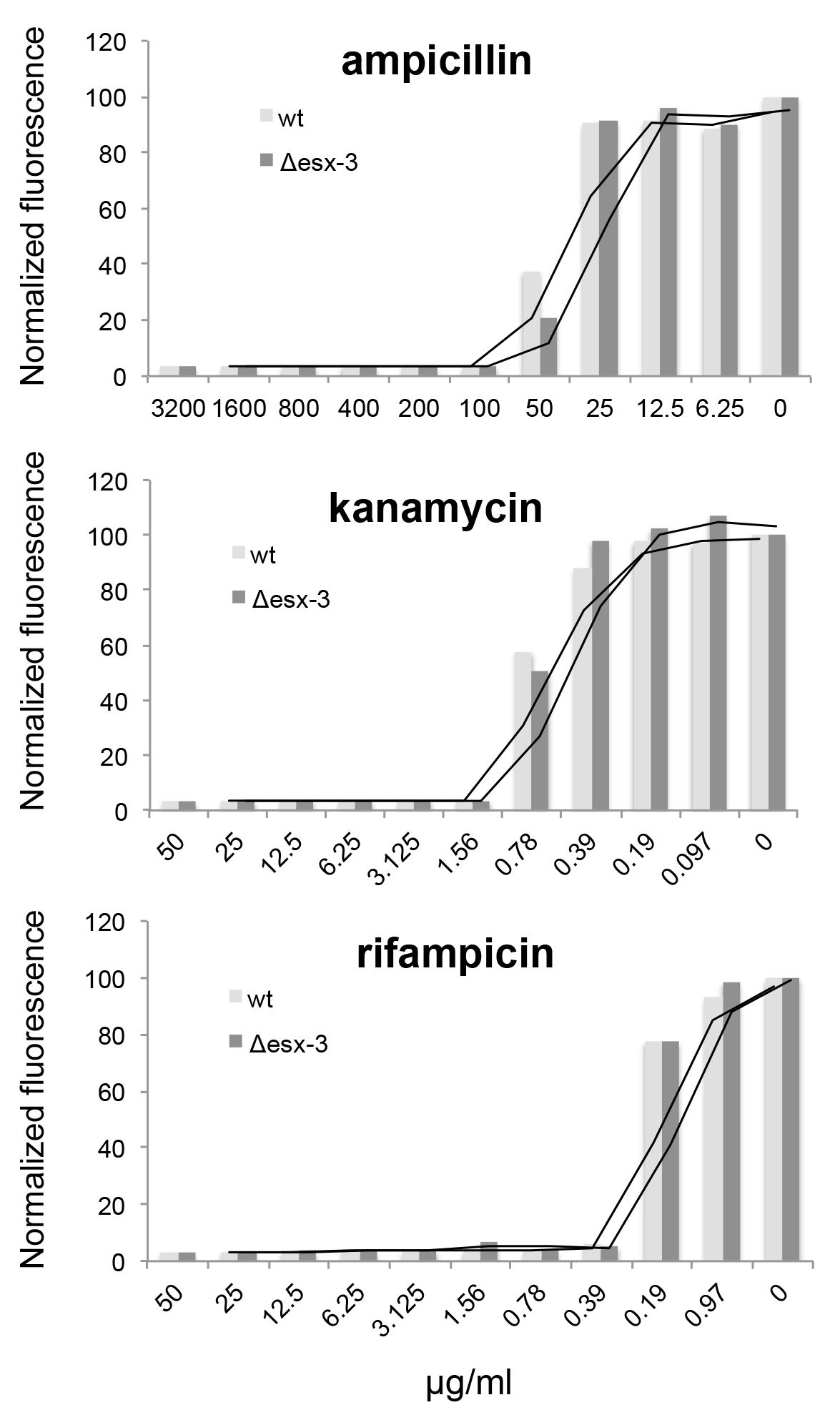

Supplement: Figure S5 — Determination of the MIC to different antibiotics in the M. smegmatisesx-3 null mutant. The y-axes reports the normalized fluorescence signal from Alamar blue dye, while the x-axes indicates antibiotic concentrations. The fluorescent signal was normalized respect to the fluorescence obtained from cultures not treated with drugs. The trend-lines are shown. (TIF) [file pone.0078351.s005.tif]

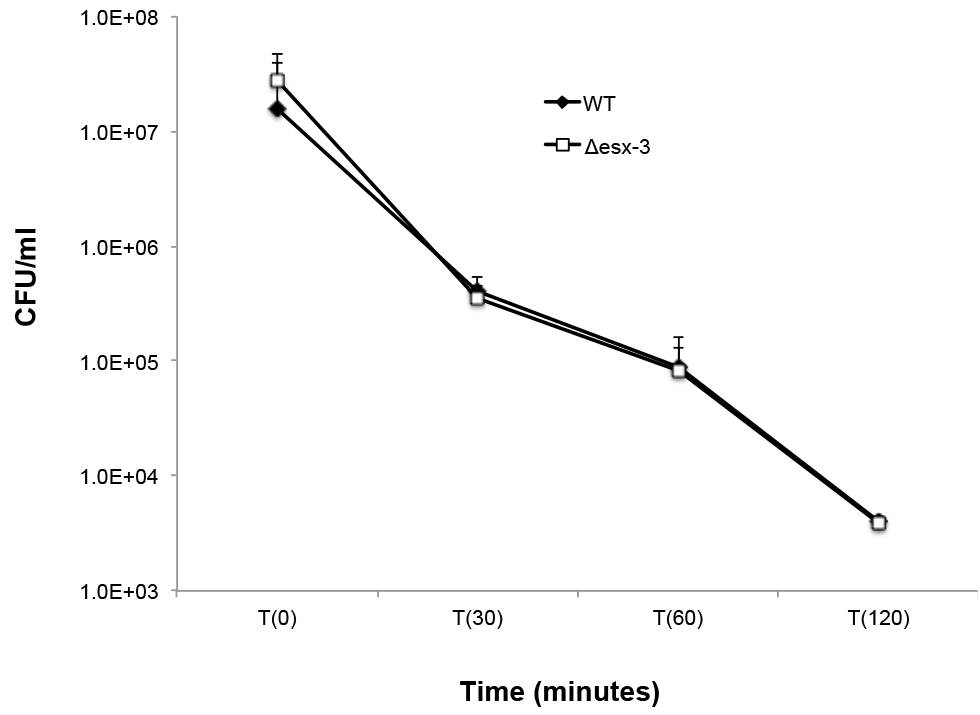

Supplement: Figure S6 — Survival of the M. smegmatisesx-3 null mutant after exposure to 0.1% SDS. The experiment, plated in triplicate, was repeated twice using independent mycobacterial cultures. Values represent the average and the error standard obtained for each point in one representative experiment. (TIF) [file pone.0078351.s006.tif]
