# Supplementary material for: The ESX-3 Secretion System Is Necessary for Iron and Zinc Homeostasis in Mycobacterium tuberculosis
Source: PLoS One. 2013 Oct 14;8(10):e78351. doi: 10.1371/journal.pone.0078351 (PMC3796483; doi:10.1371/journal.pone.0078351)
Supplement: Table S1 — List of the strains and plasmids used and constructed in this study. (PDF) [file pone.0078351.s007.pdf]

Table S1. List of the strains and plasmids used and constructed in this study.

Strains	Genotype	References
M. tuberculosis		
TB79<br>TB38	Conditional mutant *Pptr::esx-3* derived by TB38<br>TetR-Pip system in L5 *attB* site derived by H37Rv	Serafini, et al. 2009<br>Boldrin et al. 2010
M. smegmatis		
mc^2 155<br>MS190<br>MS192<br>MS195<br>MS197<br>MS81.1	Wild type<br>*esx-3* null mutant<br>*esx-3* null mutant with pJV53 plasmid<br>*esx-3/fxbA* null mutant<br>*fxbA* null mutant<br>mc^2 155 with pJV53 plasmid	Laboratory stock<br>This study<br>This study<br>This study<br>This study<br>Laboratory stock
Plasmids		
p2NIL	Suicide vector for mycobacteria OriE (Kan^R)	Parish and Stoker, 2000
pGOAL19	OriE; *LacZ SacB* genes; (Amp^R; $Hygro^R$)	Parish and Stoker, 2000
pAL70	OriE; *dif* cassette ($Hygro^R$)	Cascioferro et al., 2010
pJV53	Replicative vector for mycobacteria. OriE; OriM; recombinant protein gp60-gp61 of the Che9c mycobacteriophage (Kan^R)	Van Kessel and Hatfull, 2007
pCR-BluntII-TOPO	OriE (Zeo^R; Kan^R)	Invitrogen
pAGN25	Suicide vector derived from p2NIL OriE (Kan^R)	This study
pAGN27	Suicide vector derived by pAGN25 OriE; *LacZ SacB* genes; (Amp^R; $Hygro^R$)	This study
pAGN29	Derived by pCR-BluntII-TOPO OriE (Zeo^R; Kan^R)	This study
pAGN30	Derived by pAGN29 OriE *LacZ SacB* (Zeo^R; Kan^R $Hygro^R$)	This study

-Boldrin F, Casonato S, Dainese E, Sala C, Dhar N, et al. (2010) Development of a repressible mycobacterial promoter system based on two transcriptional repressors. Nucleic Acids Res 38:e134.

-Cascioferro A, Boldrin F, Serafini A, Provvedi R, Palu G, et al. (2010) Xer site-specific recombination, an efficient tool to introduce unmarked deletions into mycobacteria. Appl Environ Microbiol 76: 5312-5316.

-Parish T, Stoker NG (2000) Use of a flexible cassette method to generate a double unmarked Mycobacterium tuberculosis tlyA plcABC mutant by gene replacement. Microbiology 146: 1969-1975.

-Serafini A, Boldrin F, Palu G, Manganelli R (2009) Characterization of a *Mycobacterium tuberculosis* ESX-3 conditional mutant: essentiality and rescue by iron and zinc. J Bacteriol 191: 6340-6344.

-van Kessel JC, Hatfull GF (2007) Recombineering in *Mycobacterium tuberculosis*. Nat Methods 4: 147-152.
