# Supplementary material for: The ESX-3 Secretion System Is Necessary for Iron and Zinc Homeostasis in Mycobacterium tuberculosis
Source: PLoS One. 2013 Oct 14;8(10):e78351. doi: 10.1371/journal.pone.0078351 (PMC3796483; doi:10.1371/journal.pone.0078351)
Supplement: Table S2 — List of primers used in this study for mutants construction. (PDF) [file pone.0078351.s008.pdf]

Table S2. List of primers used in this study for mutants construction. The restriction sites are indicated in bold.

**Name**	**Sequence**
RP1215 (Fw)	**GGATCC**GTCGACCTTCGTGATGACTG
RP1216 (Rv)	**ATCGAT**GCATAGGCAAGTTGGAAGTC
RP1217 (Fw)	**ATCGAT**GCGTCATCAACATCTCACTG
RP1218 (Rv)	**AGTACT**GTGGTCAGGAACATGCCTTT
RP1258 (Fw)	GAACTGGGCTTCACCTAC
RP1279 (Rv)	CCTGTCCAGATAACCGACCA
RP1277 (Fw)	GAGTTCGGTGTGGACTTCGT
RP1278 (Rv)	GACCTGCCATCGTGAAACTC
RP1261 (Fw)	GCTATGCATC**AAGCTT**ACTTGAGATTTCGGCTCACG
RP1262 (Rv)	GGTAGCGAAT**AGATCT**CACAGGAAACACCCAGCTTT
RP1263 (Fw)	ATTCGCCCTT**AGATCT**ATTCGCTACCGTTTGACCAC
RP1264 (Rv)	AATTGGGCCC**TCTAGA**TTTTCCGCATCAGAAGGTGT
RP472 (Rv)	GGCTTGGTGGACCTCGAC
RP747 (Fw)	CGCCTTCACCTTCCTGCACGACTT
RP1308 (Fw)	TAGACCACGGTCAACACCAC
RP1309 (Rv)	TGACTCACACGCCAGAGAAC
