# Supplementary material for: The ESX-3 Secretion System Is Necessary for Iron and Zinc Homeostasis in Mycobacterium tuberculosis
Source: PLoS One. 2013 Oct 14;8(10):e78351. doi: 10.1371/journal.pone.0078351 (PMC3796483; doi:10.1371/journal.pone.0078351)
Supplement: Table S3 — List of the primers used in for real time RT-PCR. (PDF) [file pone.0078351.s009.pdf]

Table S3. List of the primers used in for real time RT-PCR.

Name	Sequence	Gene name
RP1070 (Fw)	GCTGTCGGATACCAGCTTCG	*rv0282*
RP1071 (Rv)	GACACCTGGCGTTTGACCTC	
RP1516 (Fw)	CCATCCCGAAAAGGAAGACC	*sigA*
RP1517 (Rv)	AGGTCTGGTTCAGCGTCGAG	
RP1163 (Fw)	ACTCGATCCGGATGATGTCG	*mbtB*
RP1164 (Rv)	GCTCGCCTTCCTGGGATAAC	
RP1165 (Fw)	GGGTTGCAGGGTGTGATGTT	*irtA*
RP1166 (Rv)	GCTGGAACTCGGTGTTGGAC	
RP1399 (Fw)	AAAATCCCGGCGGTCTCTAC	*rv1460*
RP1400 (Rv)	GATCAGCGCGTCCAGATGAC	
RP1401(Fw)	CAGGGGTCGATCCCAAGAAT	*rv0097*
RP1402 (Rv)	GATCTCCGGGTGGTCTTCGT	
RP131(1)	GTCCGCCCACTGCCAAG	*rpmB2*
RP132(1)	GGACACCCGCAGCCGAA	
RP288(1)	ACCGCCGCCGTGACCGC	*rv2059*
RP289(1)	CGGGGGTGGTGGTCGGG	

(1) Maciag, A., E. Dainese, G. M. Rodriguez, A. Milano, R. Provvedi, M. R. Pasca, I. Smith, G. Palu, G. Riccardi, and R. Manganelli. 2007. Global Analysis of the Mycobacterium tuberculosis Zur (FurB) Regulon. J Bacteriol 189:730-740.
