# Supplementary material for: The ESX-3 Secretion System Is Necessary for Iron and Zinc Homeostasis in Mycobacterium tuberculosis
Source: PLoS One. 2013 Oct 14;8(10):e78351. doi: 10.1371/journal.pone.0078351 (PMC3796483; doi:10.1371/journal.pone.0078351)
Supplement: Table S4 — Differentially regulated genes in ESX-3-depleted M. tuberculosis. (PDF) [file pone.0078351.s010.pdf]

Table S4. Differentially regulated genes in ESX-3-depleted *M. tuberculosis*

Upregulated genes					
Locus	Gene Name	*q*-value	Fold induction	Gene Product/Function	Regulation
ORF01283		0	12.96	Hypothetical ORF	
ORFD1079		0	7.33	Hypothetical ORF	
ORF00540		0	2.1	Hypothetical ORF	
Rv0097		0	5.3 (15)	Possible oxidoreductase, predicted to be in SenX3-RegX3 regulon	
Rv0099	*fadD10*	0.75	7.57	Possible fatty-acid-CoA ligase	
Rv0146		0	2.48	Possible methyltransferase S-adenosylmethinonine dependent	
Rv0211	*pckA*	0	2.49	Probable iron-regulated phosphoenolpyruvate carboxykinase	
Rv0280	PPE3	0	8.99	Unknown function	Zur
Rv0281		0.75	2.8	Possible S-adenosylmethionine dependent methyl transferase	Zur
Rv0450c	*mmpL4*	0.64	2.01	Siderophore export protein	IdeR
Rv0451c	*mmpS4*	0.65	2.29	Siderophore export protein	IdeR
Rv0465c		0	2.37	Probable transcriptional regulatory protein	IdeR-independent iron repressed
Rv0467	*icl1*	0	3.32	Isocitrate lyase	IdeR-independent iron repressed
Rv0468	*fadB2*	0	1.82	Involved in the butyryl-butanol producing pathway/Probable 3-hydroxybutyryl-CoA dehydrogenase	
Rv1342c	*pks14*	0	2.31	Conserved membran protein	
Rv1343c	*lprD*	0	2.27	Probable conserved lipoprotein	IdeR
Rv1344	*mbtL*	0	2.28	Thought to be involved in the biogenesis of the hydroxyphenyloxaoline-containing siderophore mycobactins	IdeR
Rv1347c	*mbtK*	0	3.92	Lysine N-acetyltransferase/ Thought to be involved in the biogenesis of the hydroxyphenyloxaoline-containing siderophore mycobactins/	IdeR
Rv1348	*irtA*	2.22	4.78 (5.4)	Iron regulated transporter	IdeR
Rv1349	*irtB*	3.78	2.39	Iron regulated transporter	IdeR
Rv1460		0	2.32 (2.4)	Probable transcriptional regulatory protein	IdeR-independent iron repressed
Rv1461		0	2.79	Conserved hypothetical protein	IdeR-independent iron repressed
Rv1462		0.82	2.44	Conserved hypothetical protein	IdeR-independent iron repressed
Rv1463		0	2.62	Probable ABC transporter	IdeR-independent iron repressed
Rv1464	*csd*	0	2.61	Probable cysteine desulfurase	IdeR-independent iron repressed
Rv1465		0.72	2.06	Possible nitrogen fixation related protein	IdeR-independent iron repressed
Rv1466		0	2.59	Conserved hypothetical protein	IdeR-independent iron repressed
Rv1519		0	2.96	Conserved hypothetical protein	IdeR

Rv1592c		0	2.1	Conserved hypothetical protein	
Rv1854	*ndh*	0	2.03	NADH dehydrogenase	
Rv2055c	*rpsR2*	0	12.39	Probable ribosomal protein S18/ involved in translation	Zur
Rv2056c	*rpsN2*	0	15.76	Probable ribosomal protein S14/ involved in translation	Zur
Rv2057c	*rpmG*	0	17.67	Probable ribosomal protein L33/ involved in translation	Zur
Rv2058c	*rpmB2*	0	15.13 (9.8)	Probable ribosomal protein L28/ involved in translation	Zur
Rv2059		0	2.73 (3.9)	Similar to periplasmic metal binding proteins of ABC transport system	Zur
Rv2060		1,19	2.53	Similar to ZnuB, ABC-type Mn2/Zn2 transport systems,permease components	Zur
Rv2121c	*hisG*	2,01	2.09	Thought to be involved in histidine biosynthesis/ Probable ATP phosphoribosyltransferase	
Rv2122c	*hisI/hisE*	0	5.13	Probable phosphoribosyl-AMP pyrophosphatase/ thought to be involved in histidine biosynthesis	IdeR
Rv2123	PPE37	0	10.75	PPE family	IdeR
Rv2377c	*mbtH*	0,8	3.68	Thought to be involved in the biogenesis of the hydroxyphenyloxaoline-containing siderophore mycobactins	IdeR
Rv2380c	*mbtE*	2.01	5.08	Thought to be involved in the biogenesis of the hydroxyphenyloxaoline-containing siderophore mycobactins	IdeR
Rv2381c	*mbtD*	0	5.92	Thought to be involved in the biogenesis of the hydroxyphenyloxaoline-containing siderophore mycobactins	IdeR
Rv2382c	*mbtC*	0	7.51	Thought to be involved in the biogenesis of the hydroxyphenyloxaoline-containing siderophore mycobactins	IdeR
Rv2383c	*mbtB*	0	8.6 (27.1)	Thought to be involved in the biogenesis of the hydroxyphenyloxaoline-containing siderophore mycobactins	IdeR
Rv2384c	*mbtA*	2.47	2.4	Thought to be involved in the biogenesis of the hydroxyphenyloxaoline-containing siderophore mycobactins	IdeR
Rv2386c	*mbtI*	0	21.43	Thought to be involved in the biogenesis of the hydroxyphenyloxaoline-containing siderophore mycobactins	IdeR
Rv2429	*ahpD*	0	4.22	Involved in response to oxidative stress/Alkyl hydroperoxide reductase D protein	
Rv2707		0	2.07	Conserved transmembrane alanine and leucine rich protein	
Rv3403c		3.9	1.9	Hypothetical protein	IdeR
Rv3839		0	9.88	Conserved hypothetical protein	IdeR
Rv3840		0	2.99	Probable transcriptional regulatory protein	IdeR
Rv3862c	*whiB6*	0	2.19	Probable transcriptional regulatory protein whiB-like	
**Down regulated genes**					
Locus	Gene Name	*q*-value	Fold repression	Gene Product/Function	Regulation
ORFD0126		0	0.57	Hypothetical ORF	
Rv0165c	*mce1R*	0	0.65	Probable transcriptional regulatory protein	
Rv0166	*fadD5*	0	0.51	Involved in lipid degradation/probable fatty-acid-CoA ligase	
Rv0283	*eccB3*	0	0.25	Esx-3 component/Membrane protein	IdeR/Zur
Rv0285	PE	2.17	0.27	Esx-3 component/PE5	IdeR/Zur
Rv0289	*eccG3*	0.88	0.24	Esx-3 component/ESX-1 secreted associated protein ESPG3	IdeR/Zur
Rv0290	*eccD3*	0	0.19	Esx-3 component/Transmembrane protein	IdeR/Zur
Rv0291	*mycP3*	0	0.27	Esx-3 component/Serine protease (subtilisin-like protease)	IdeR/Zur

Rv0292	*eccE3*	0	0.24	Esx-3 component/Transmembrane protein	IdeR/Zur
Rv0458		0	0.61	Probable aldheyde dehydrogenase	
Rv1813c		0	0.49	Conserved hypothetical protein/ regulated by MprA and DosR	
Rv3145	*nuoA*	0	0.34	Involved in aerobic-anaerobic respiration/probable NADH dehydrogenase I (chain A)	Iron induced
Rv3147	*nuoC*	0	0.48	Involved in aerobic-anaerobic respiration/probable NADH dehydrogenase I (chain C)	Iron induced
Rv3148	*nuoD*	5.58	0.42	Involved in aerobic-anaerobic respiration/probable NADH dehydrogenase I (chain D)	Iron induced
Rv3149	*nuoE*	0	0.48	Involved in aerobic-anaerobic respiration/probable NADH dehydrogenase (chain E)	Iron induced
Rv3151	*nuoG*	0	0.59	Involved in aerobic-anaerobic respiration/probable NADH dehydrogenase (chainG)	Iron induced
Rv3152	*nuoH*	0.88	0.54	Involved in aerobic-anaerobic respiration/probable NADH dehydrogenase (chain H)	Iron induced
Rv3153	*nuoI*	0	0.55	Involved in aerobic-anaerobic respiration/probable NADH dehydrogenase (chain I)	Iron induced
Rv3154	*nuoJ*	0	0.6	Involved in aerobic-anaerobic respiration/probable NADH dehydrogenase (chain J)	Iron induced
Rv3156	*nuoL*	5.46	0.59	Involved in aerobic-anaerobic respiration/probable NADH dehydrogenase (chain L)	Iron induced
Rv3157	*nuoM*	2.46	0.52	Involved in aerobic-anaerobic respiration/probable NADH dehydrogenase (chain M)	Iron induced
Rv3158	*nuoN*	1.14	0.67	Involved in aerobic-anaerobic respiration/probable NADH dehydrogenase (chain N)	Iron induced
Rv3295		0	0.47	Probable transcriptional regulatory protein (Tet-R family)	
Rv3550	*echA20*	0	0.6	Enoyl-CoA hydratase	

Differently expressed genes were defined by a *q*-value equal to 0 and a fold difference ≥1.8 (induced) or ≤0.6 (repressed). Differentially regulated genes with q-value until 5,58 were included if part of an operon or regulon differentially regulated or if their up regulation was observed by real time RT-PCR (i.e. *irtA* and *irtB).* Values in brackets indicate the fold induction determined by quantitative real time RT-PCR. Genes were annotated as described in TUBERCULIST http://tuberculist.epfl.ch/
